# Sox10 is required for systemic initiation of bone mineralization

**DOI:** 10.1242/dev.204357

**Published:** 2025-01-20

**Authors:** Stefani Gjorcheska, Sandhya Paudel, Sarah McLeod, David Paulding, Louisa Snape, Karen Camargo Sosa, Cunming Duan, Robert Kelsh, Lindsey Barske

**Affiliations:** ^1^ Division of Human Genetics, Cincinnati Children's Hospital Medical Center, Cincinnati, OH 45229, USA; ^2^ Department of Life Sciences, University of Bath, Bath BA2 7AY, UK; ^3^ Department of Molecular, Cellular, and Developmental Biology, University of Michigan, Ann Arbor, MI 48109, USA; ^4^ Department of Pediatrics, University of Cincinnati College of Medicine, Cincinnati, OH 45229, USA

**Keywords:** Sox10, Bone mineralization, Neural crest, Calcium, Stanniocalcin, Zebrafish

## Abstract

Heterozygous variants in *SOX10* cause congenital syndromes affecting pigmentation, digestion, hearing, and neural development, primarily attributable to failed differentiation or loss of non-skeletal neural crest derivatives. We report here an additional, previously undescribed requirement for Sox10 in bone mineralization. Neither crest- nor mesoderm-derived bones initiate mineralization on time in zebrafish *sox10* mutants, despite normal osteoblast differentiation and matrix production. Mutants are deficient in the Trpv6^+^ ionocytes that take up calcium from the environment, resulting in severe calcium deficiency. As these ionocytes derive from ectoderm, not crest, we hypothesized that the primary defect resides in a separate organ that systemically regulates ionocyte numbers. RNA sequencing revealed significantly elevated stanniocalcin (Stc1a), an anti-hypercalcemic hormone, in *sox10* mutants. Stc1a inhibits calcium uptake in fish by repressing *trpv6* expression and Trpv6^+^ ionocyte proliferation. Epistasis assays confirm excess Stc1a as the proximate cause of the calcium deficit. The pronephros-derived glands that synthesize Stc1a interact with *sox10*^+^ cells, but these cells are missing in mutants. We conclude that *sox10*^+^ crest-derived cells non-autonomously limit Stc1a production to allow the inaugural wave of calcium uptake necessary to initiate bone mineralization.

## Teaser

**Summary:** *sox10*-dependent neural crest cells restrain production of anti-hypercalcemic hormones to enable the massive influx of calcium needed to initiate bone mineralization in developing fish.

## INTRODUCTION

Sry-box transcription factor 10 (SOX10) is essential for hair and skin pigmentation, the ability to hear and smell, and for digestive peristalsis. People with only one functional copy of the *SOX10* gene present pigment anomalies such as iris heterochromia and a white forelock, sensorineural hearing loss, deficient enteric innervation, anosmia, neurological abnormalities, neuropathy, and/or stalled puberty. Cases range from mild to potentially lethal and are classified into four congenital syndromes with overlapping clinical features: Waardenburg syndrome types 2E and 4C, Kallmann syndrome, and PCWH (peripheral demyelinating neuropathy, central dysmyelination, Waardenburg syndrome, and Hirschsprung disease) (Pingault et al., 2022). Besides the inner ear and central nervous system phenotypes, these symptoms are largely attributable to failed neural crest differentiation. This transient, migratory population of embryonic cells gives rise to pigment cells, sensory and enteric neurons and glia, the adrenal medulla, and bone, cartilage, and connective tissues of the facial skeleton (Martik and Bronner, 2017). All neural crest cells (NCCs) activate *SOX10* expression upon specification, prior to migration. The cranial subpopulation destined to give rise to the facial skeleton turn *SOX10* expression off upon reaching their destination in the pharyngeal arches (Kuhlbrodt et al., 1998; Blentic et al., 2008). The remaining, non-skeletal neural crest populations retain Sox10 expression longer to activate programs for differentiation into pigment, glia, and sensory or enteric neurons, among other cell types (Antonellis et al., 2008); in mutants, migration and differentiation stall, and many of these cells die (Dutton et al., 2001). Sox10 is also expressed in differentiating chondrocytes of both neural crest and mesodermal origin, but it is not essential there, as cartilage develops normally in zebrafish *sox10* mutants (Kelsh and Eisen, 2000). Accordingly, decades of research on heterozygous humans as well as homozygous mouse and zebrafish models has established that SOX10 is essential for non-skeletal neural crest derivatives but likely dispensable for formation of the skeleton (Herbarth et al., 1998; Southard-Smith et al., 1998; Kelsh and Eisen, 2000; Britsch et al., 2001; Dutton et al., 2001).

Bones mineralize by packing an organic collagenous extracellular matrix (ECM) with hydroxyapatite crystals of calcium and phosphate in a highly ordered manner (Blair et al., 2002). Mature bone-forming osteoblasts secrete collagen I/X-rich ECM as well as enzymes (e.g. alkaline phosphatase, Phospho1) and accessory glycoproteins (e.g. osteopontin, osteonectin) involved in synthesis and organization of the hydroxyapatite crystals (Bianco et al., 1988; Yamate et al., 1997; Millan, 2013). Failed osteoblast maturation, disturbed matrix formation, and calcium-phosphate imbalances can disrupt ossification (Michigami, 2019; Ponzetti and Rucci, 2021).

Endocrine factors, particularly parathyroid hormone, vitamin D, and calcitonin, work in concert to maintain calcium and phosphate homeostasis in adults through actions on bone, intestine, and kidney (Waung et al., 2012; Guo and Yuan, 2015; Khundmiri et al., 2016; Areco et al., 2020; Hanna et al., 2021). Adults obtain these minerals from dietary sources, renal reabsorption, and environmental uptake, as well as by breaking down bone (Wongdee et al., 2019). However, how the initial wave of calcium and phosphate uptake in the embryo is regulated remains a gap in knowledge. Mammalian fetuses rely on placental transfer (Kovacs, 2014), while fish larvae utilize yolk stores or ionocytes in the skin and gills for environmental uptake (Hwang and Chou, 2013; Lin and Hwang, 2016). Although zebrafish larvae obtain the necessary amount of phosphate through phospholipid metabolism in the yolk and do not require additional environmental phosphate uptake (Miyares et al., 2014; Fraher et al., 2016; Quinlivan and Farber, 2017), they require calcium uptake for skeletal mineralization (Vanoevelen et al., 2011). The major route of calcium ingress is the constitutively open epithelial calcium channel (ECaC) encoded by *trpv6* (Transient Receptor Potential channel family, Vanilloid subfamily member 6) (Khattar et al., 2022). Of the five major types of ionocytes in fish, only the Na^+^/H^+^-ATPase-rich (NaR) subpopulation expresses *trpv6* (Hwang and Chou, 2013; Xin et al., 2019). *Trpv6* expression is also highly enriched in both maternal and fetal cells of the mammalian placenta (Fecher-Trost et al., 2019). Because Trpv6 is constitutively open, regulation of calcium uptake occurs through modulation of *trpv6* transcription or the proliferation/quiescence of *trpv6*^+^ cells (Xin et al., 2019). Whether the major endocrine hormones involved in calcium and phosphate homeostasis in adults also control the initiation of calcium uptake via Trpv6 for skeletal mineralization in the embryo remains largely unknown.

One factor that limits rather than drives calcium uptake in both embryonic and adult fish is an anti-hypercalcemic hormone called stanniocalcin (Tseng et al., 2009). Stanniocalcin (Stc1) is a glycoprotein secreted by a variety of tissues in mammals (e.g. kidney, intestine), where it may regulate local calcium homeostasis (Madsen et al., 1998; Yoshiko and Maeda, 1998; Deol et al., 2001; Kobayashi et al., 2009; Yeung et al., 2012). Stc1a (also known as Stc1l) was first isolated from the corpuscles of Stannius (CS), intermediate mesoderm-derived endocrine organs unique to teleost fish (Pang et al., 1973; Yeung et al., 2012; Naylor et al., 2018). Surgical removal of the CS or mutation of the *stc1a* gene (also known as *stc1l*) causes severe hypercalcemia, kidney stones, and increased NaR cell numbers in fishes (Fenwick and So, 1974; Yeung et al., 2012; Li et al., 2021). Conversely, exposure to high environmental calcium increases *stc1a* mRNA levels and serum Stc1a content in zebrafish, leading to decreased calcium uptake (Tseng et al., 2009). The anti-hypercalcemic activity of Stc1a involves inhibition of both *trpv6* expression and NaR cell proliferation, working through a Pappaa-Igfbp5a-Igf-Igfr cascade that impacts PI3K, mTor, and Akt signaling. In normal or low calcium, active Pappaa cleaves the Igf-binding protein Igfbp5a, releasing Igf ligands to activate downstream signaling and NaR cell proliferation. In conditions of high environmental calcium, Stc1a inhibits the protease activity of Pappaa, keeping NaR cells quiescent (Dai et al., 2014; Oxvig, 2015; Liu et al., 2020; Li et al., 2021, 2023).

In this study, we present a previously undescribed systemic requirement for Sox10 in the initiation of skeletal mineralization in fish. We provide evidence of a striking Stc1a increase in *sox10* mutants that severely reduces *trpv6*^+^ ionocyte number and whole-body calcium content. We find *sox10*^+^ neural crest-derived cells interacting with the CS in control but not mutant fish, indicating that they may serve to moderate Stc1a levels in the embryo, allowing the massive wave of calcium uptake required to initiate bone mineralization.

## RESULTS

### Delayed onset of skeletal mineralization in zebrafish *sox10* mutants

**Fig. 1.**
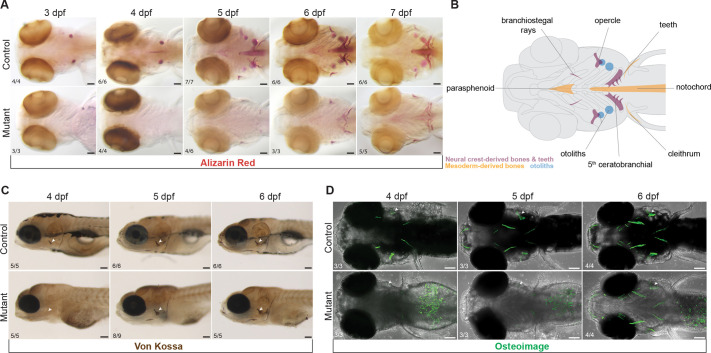
**Mineralization deficit in zebrafish *sox10* mutants.** (A) Example images illustrating a major delay in initiation of bone mineralization in *sox10* mutants between 3 and 7 dpf revealed by Alizarin Red staining. Some mineralization is present by 5 dpf but never achieves control levels before lethality at 8 dpf. (B) Schematic representation of the affected mineralized structures and their embryonic origins. (C,D) Von Kossa (C) and OsteoImage (D) staining showing absent calcium deposition and hydroxyapatite formation in *sox10* mutants at 4 dpf and gradual recovery starting at 5 dpf. Arrowheads point to the opercle (op). Numbers in panels indicate the proportion of larvae of that genotype with the presented phenotype. Scale bars: 100 µm.

Although Sox10 expression is activated in all NCCs upon specification (Dutton et al., 2001; Antonellis et al., 2008; Stine et al., 2009; Kague et al., 2012), it is quickly downregulated in the crest cells that form the facial skeleton (Blentic et al., 2008). It was assumed that these skeletal progenitors do not require Sox10 function, as early studies noted no defects in Alcian Blue-labeled cartilages of the zebrafish *sox10* mutant facial skeleton (Kelsh and Eisen, 2000). However, during a routine bone stain of *sox10* mutants, we unexpectedly noticed a striking absence of mineralization when the skeleton is first differentiating at 3-4 days post-fertilization (dpf) (Fig. 1A). At these stages, calcium deposits in newly mineralizing bones were readily apparent by Alizarin Red staining in sibling controls. Weak staining appeared in mutants by 5 dpf and increased until larval lethality around 8 dpf, but never attained control levels. The phenotype, unrelated to edema or developmental delay, was indiscriminate of ossification type (endochondral, intramembranous, and even odontogenic) and bone developmental origin in the mesoderm versus neural crest (Fig. 1B).

As deficient mineralization had not been reported in any of the many existing mouse, fish, or frog Sox10 loss-of-function models, we questioned whether it is a neomorphism specific to our *sox10^ci3020^* allele. *ci3020* is a 1495-bp deletion that removes part of the 5′UTR and the first coding exon, encoding the homodimerization domain and part of the DNA-binding high mobility group (HMG) domain (Fig. S1A). Some transcription still occurs from the deletion allele (Okeke et al., 2022), and the first in-frame methionine downstream of the deletion could conceivably produce an N-terminally truncated protein lacking the HMG box but retaining the transactivation domain (Pingault et al., 2022). *sox10^ci3020/ci3020^* embryos otherwise present the classic *colourless* phenotypes associated with *sox10* loss of function (Fig. S1B-C′) (Kelsh et al., 1996), lacking melanocytes and xanthophores, with malformed otic vesicles and otoliths but normal facial cartilages. To test whether deficient mineralization is specific to the *ci3020* allele, we performed Alizarin Red staining on homozygotes for the *m618* (L142Q) missense allele (Malicki et al., 1996). The same near-absence of staining was observed between 4 and 6 dpf (Fig. S1D), demonstrating that this phenotype is a general consequence of loss of *sox10* function, at least in zebrafish. We further validated the Alizarin Red results in *ci3020* mutants (hereafter *sox10* mutants) using Von Kossa and calcein stains (Fig. 1C, Fig. S1C′), which both label calcium deposits (Puchtler and Meloan, 1978; Du et al., 2001; Teng et al., 2018; Schneider, 2021), as well as the OsteoImage mineralization assay (Fig. 1D), which specifically detects hydroxyapatite (Sim et al., 2018). These stains confirmed that mineralization gradually initiates around 5 dpf, first apparent by Von Kossa staining (Fig. 1C). Supporting that the recovery is incomplete, fluorescent calcein staining in older 7 dpf larvae revealed a lack of endochondral bone collars around the mutant hyomandibula and ceratohyal cartilages (Fig. S1C′).

**Fig. 2.**
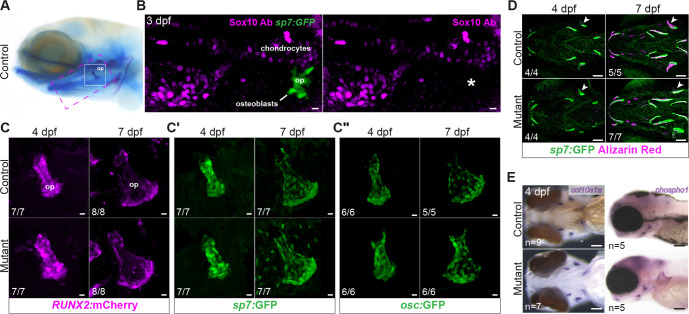
**Normal patterns of growth and differentiation in *sox10* mutant osteoblasts.** (A) Reference image of a larva stained with Alcian Blue and Alizarin Red, with locations of the skeletal elements shown in B (magenta dashed box) and C (white box) highlighted. (B) Immunostaining with an anti-Sox10 antibody reveals strong expression in chondrocytes but a lack of Sox10 protein (asterisk) in mineralizing osteoblasts (*sp7*:GFP^+^) forming the op bone at 3 dpf. (C-C″) Example images from sequential live imaging showing normal patterns of *RUNX2*:mCherry, *sp7*:GFP and *osc*:GFP transgene expression in mutant osteoblasts of the op at 4 and 7 dpf. (D) Normal growth of *sox10* mutant op (arrowheads) as well as other bones despite minimal calcium accumulation, revealed by live imaging of Alizarin Red-stained *sp7*:GFP^+^ embryos at 4 and 7 dpf. (E) Colorimetric *in situ* hybridizations for *col10a1a* and *phospho1*, encoding key bone matrix components, revealed no overt abnormalities in *sox10* mutants at 4 dpf. op, opercle. Numbers in panels indicate the proportion of larvae of that genotype with the presented phenotype. Scale bars: 10 µm (B-C″); 100 µm (D,E).

Osteoblasts are essential for mineralization, but do not themselves express Sox10 (Fig. 2A,B) (Gomez-Picos et al., 2022). Osteoblasts derived from cranial neural crest transiently express *sox10* and accordingly express the *SOX10:*Cre neural crest lineage label (made with a human neural crest-specific *SOX10* enhancer) (Antonellis et al., 2008; Kague et al., 2012), but mesoderm-derived osteoblasts never pass through a *sox10*^+^ state. We therefore presumed that the broad mineralization deficit would not be cell-autonomous to osteoblasts, although it was possible that their differentiation could be impacted by extrinsic factors. Osteoblasts are evident as early as 3 dpf at the site of the future opercle (op) bone (Stenzel et al., 2022). To evaluate mutant osteoblasts, we used the established transgenic markers *RUNX2*:mCherry (Barske et al., 2020), *sp7*:EGFP (DeLaurier et al., 2010) and *osc*:EGFP (Vanoevelen et al., 2011), which are respectively activated in osteoprogenitors and early and maturing osteoblasts. Live imaging of the op bone in *sox10* mutants and sibling controls from 3 to 7 dpf revealed seemingly normal patterns for each marker (Fig. 2C-C″). Visualization of *sp7:*GFP in combination with live Alizarin Red staining confirmed that individual elements are growing similarly between mutants and controls (Fig. 2D). Colorimetric *in situ* hybridization for the major bone ECM component *col10a1a* also revealed normal expression in mutants (Fig. 2E). These findings suggest that mutant osteoblasts are still differentiating and making collagenous matrix despite not being able to mineralize it.

To determine whether the mineralization machinery is intact in *sox10* mutant osteoblasts, we performed *in situ* hybridization and/or semi-quantitative reverse-transcriptase PCR (rt-PCR) for *spp1* (Osteopontin), *sparc* (Osteonectin), *alpl* (Alkaline phosphatase), *enpp1*, *entpd5*, *phex*, *fgf23*, *runx2a*, and *runx2b* (Fig. 2E, Fig. S2)*.* These genes encode secreted proteins and enzymes associated with matrix formation, phosphate and calcium regulation, and hydroxyapatite synthesis, in addition to the Runx2 transcription factors required for osteoblast specification. *In situ* hybridizations revealed unchanged *runx2a* and *runx2b* expression (Fig. S2A), aligning with the *RUNX2:*mCherry live imaging (Fig. 2D). *phospho1* expression appeared largely unchanged in forming mutant bones (Fig. 2E), while *sparc* and *spp1* appeared reduced (Fig. S2A). In rt-PCRs performed on cDNA made from pooled 4-dpf embryos, we detected mild increases in *alpl* and *entpd5* in the mutants (*P*<0.05, unpaired *t*-tests; Fig. S2B), but no change in *sparc* or *phex* (Fig. S2B). We also observed slight decreases in *spp1*, *phospho1*, *enpp1*, and *fgf23* in the mutants (*P*<0.05, unpaired *t*-tests; Fig. S2B), a pattern opposite to that observed in the zebrafish *enpp1* mutant, which shows increased mineralization (Apschner et al., 2014).

To complement this candidate-based approach and resolve some of the inconsistencies, we next performed unbiased whole-body bulk RNA sequencing (RNAseq) on pools of 10-15 wild-type and mutant larvae (*n*=3 each) at 4 dpf. DESeq2 analysis identified 344 significantly downregulated (≥1.25-fold; FDR-adjusted *P*≤0.05) and 55 significantly upregulated genes in mutants (Table S1). As expected, the downregulated list is dominated by genes expressed in pigment cells (e.g. *gch2*, *defbl1*, *apoda.1*, *mc1r*) or glia/oligodendrocytes (e.g. *mbpa*, *mpz*, *cldnk*). Several collagen genes were also significantly reduced (e.g. *col1a1a*, *col1a1b*, *col1a2*), although not the osteoblast-specific *col10a1a*. *sparc* was also significantly decreased (FDR-adjusted *P*=0.0006), although none of our other osteoblast or mineralization markers met the threshold for significance (Table S2). Discrepancies across methods may reflect tissue-specific expression or differences in experimental sensitivity*.* Nevertheless, these findings collectively demonstrate for the first time that multiple factors linked with mineralization anomalies in animal models and human individuals (Yadav et al., 2011; Huitema et al., 2012a; Christov and Juppner, 2013; Si et al., 2020) may be dysregulated in *sox10* mutants.

### Mineralization deficit not associated with thyroid malfunction

Several individuals with *SOX10*-associated Kallmann syndrome exhibit altered thyroid function and reduced bone mineral density (Wang et al., 2018; Chen et al., 2020; Wakabayashi et al., 2021). The mammalian thyroid gland primarily develops from pharyngeal endoderm (De Felice and Di Lauro, 2004), with supporting cells deriving from neural crest (Le Douarin and Le Lievre, 1970). Endoderm-derived thyroid follicles secrete thyroid hormones [thyroxine (T4) and triiodothyronine (T3)], which bind to osteoblast receptors, activating pathways essential for bone mineralization and growth (Bassett and Williams, 2016). Thus, we initially hypothesized that loss of *sox10* may deplete a neural crest population important for the development of the thyroid gland, thereby reducing thyroid hormone levels and bone mineralization. To test this, we incubated *sox10* mutants and sibling controls in media containing T3 doses ranging from 50 to 600 µg l^−1^ (Fig. S3). Treated controls showed a dose-dependent reduction in melanocyte coverage (Fig. S3B), indicative of hyperthyroidism (McMenamin et al., 2014). However, no mineralization rescue was observed in 4 dpf mutants at any T3 dose (Fig. S3C). Fluorescence *in situ* hybridization showed no structural abnormalities in mutant thyroid follicles (Fig. S3A,D), located along the ventral aorta (Porazzi et al., 2009). Further, no significant differences in genes associated with thyroid hormone synthesis or signaling were uncovered in our RNAseq dataset (Table S2). These findings indicate that the mineralization deficit in zebrafish *sox10* mutants is unlikely due to thyroid dysfunction.

### *sox10* mutants are calcium deficient

**Fig. 3.**
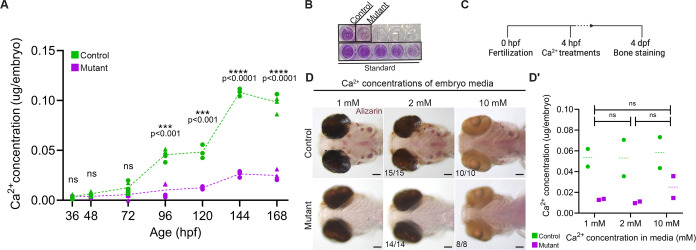
***sox10* mutants have a severe whole-body calcium deficit.** (A,B) Colorimetric calcium assay reveals significantly lower levels of Ca^2+^ in *sox10* mutants after mineralization begins at 3 dpf. Each data point represents a pool of 10-15 embryos. Different shapes represent biological replicates assayed on different days (unpaired *t*-tests; 36 hpf: *P*=0.580, d.f.=8; 48 hpf: *P*=0.083, d.f.=8; 72 hpf: *P*=0.091, d.f.=7; 96 hpf: *P*=0.0002, d.f.=6; 120 hpf: *P*=0.0008, d.f.=4; 144 hpf: *P*=0.000008, d.f.=4; 168 hpf: *P*=0.000005, d.f.=6). B shows an example of the colorimetric assay, showing a clear reduction in mutants. (C) Schematic of the Ca^2+^ treatment protocol. (D,D′) Increasing ambient Ca^2+^ levels to 2 or 10 mM does not rescue the mineralization deficit (D) or Ca^2+^ content (D′) (unpaired *t*-tests; 1 versus 2 mM: *P*=0.963, d.f.=2; 1 versus 10 mM: *P*=0.778, d.f.=2; 2 versus 10 mM: *P*=0.748, d.f.=2). Numbers in panels indicate the proportion of larvae of that genotype with the presented phenotype. In D′, ratios reflect the number of imaged larvae of that genotype with the presented phenotype, and dashed lines indicate the median. Significance determined by unpaired *t*-test. ns, not significant. Scale bars: 100 µm.

Another possible explanation for the mutant phenotype is a systemic mineral imbalance. For instance, phosphate levels are altered in fish mutant for the phosphate regulators *enpp1* and *entpd5*, impacting the expression of many other mineralization-regulating factors, including those assayed here (Huitema et al., 2012a; Apschner et al., 2014). We therefore measured calcium and phosphate levels in our mutants. Since measuring serum mineral content in larval fish is not possible, we used a colorimetric assay (Fig. 3B) on pooled whole-body samples between 36 and 168 h post-fertilization (hpf), following established protocols (Tseng et al., 2009; Suarez-Bregua et al., 2017; Li et al., 2021). In wild-type zebrafish, Ca^2+^ content began to increase around 3 dpf as the first bones mineralize and continued to rise with age (Fig. 3A) (Pan et al., 2005). By contrast, *sox10* mutants had lower Ca^2+^ content compared with controls starting at 3 dpf (*P*=0.03, unpaired *t*-test; Fig. 3A,B). Consistent with bone staining in mutants first appearing at ∼5 dpf (Fig. 1A), we found that mutant Ca^2+^ levels at 5 dpf were approximately equivalent to control levels at 3 dpf (∼0.01 µg per embryo), suggesting this may be the minimal Ca^2+^ threshold required to initiate mineralization. To investigate this possibility further, we raised wild-type embryos in medium completely devoid of Ca^2+^ and found that mineralization was absent everywhere except the otoliths inside the otic vesicles (Fig. S4B). These are made of calcium carbonate rather than hydroxyapatite (Lundberg et al., 2015; Rozycka et al., 2023) and also still form in *trpv6* mutants, which cannot take up external calcium (Vanoevelen et al., 2011; Xin et al., 2019). The Ca^2+^ content of these wild-type fish raised in 0 mM Ca^2+^ was approximately the same as that of mutants raised in 1 mM Ca^2+^ (Fig. S4A), supporting that this low level is below the threshold needed for bone mineralization. Meanwhile, phosphate levels were seemingly unaffected in mutants between 36 and 168 hpf (Fig. S4C), suggesting that lack of calcium is the major cause of the delayed and deficient hydroxyapatite formation (Fig. 1D).

Other zebrafish mutants with poor mineralization but seemingly normal osteoblasts, e.g. *msp* (*mst1*) and *trpv6* mutants, were rescued by simply increasing the concentration of Ca^2+^ in the media (Vanoevelen et al., 2011; Huitema et al., 2012b). We tested whether this would also improve our phenotype using Ca^2+^ concentrations two- and tenfold higher than our standard embryo media (2 and 10 mM versus 1 mM, respectively, following Huitema et al., 2012a,b) (Fig. 3C,D). However, Alizarin Red staining at 4 dpf revealed no increase in mineralization in mutants reared in either high-Ca^2+^ medium (Fig. 3D). We then quantified their Ca^2+^ content at 4 dpf to assess the calcium deficit specifically, finding that mutants raised in the highest Ca^2+^ environment did show a non-significant increase in Ca^2+^ content, but they remained at a severe deficit relative to controls (Fig. 3D′). Lowering or increasing the phosphate concentration likewise had no impact on mineralization in mutants (Fig. S4D). The mineralization delay in the *sox10* mutants may thus have a different etiology than other mutant lines with similar phenotypes.

### Systemic calcium dysregulation in the *sox10* mutants

**Fig. 4.**
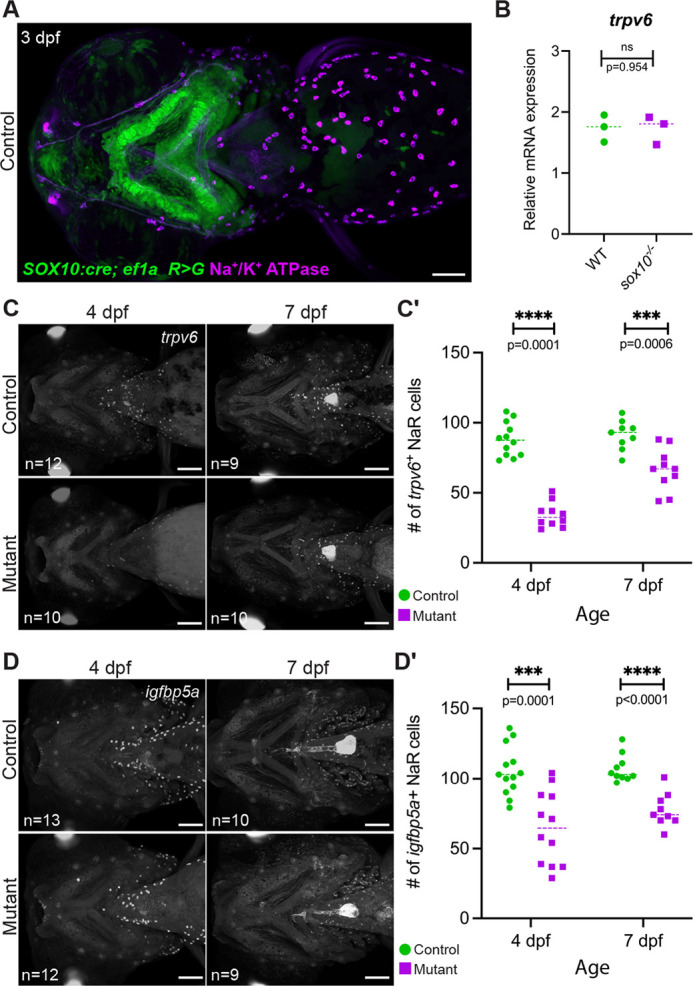
**Reduction in *trpv6*^+^ NaR cell number in the *sox10* mutants.** (A) Immunostaining of a 3 dpf *SOX10:Cre; ef1a: DsRed>GFP* larva with an antibody against the Na^+^/K^+^ ATPase pump confirms that ionocytes do not derive from neural crest. (B) rt-PCR demonstrates that *trpv6* transcription is not overtly altered at the whole-body level at 4 dpf. Each point represents a pool of 10-15 embryos (unpaired *t*-test; *P*=0.954, d.f.=4). (C-D′) Fluorescence *in situ* hybridizations for *trpv6* (C) and *igfbp5a* (D) demonstrate a striking and significant reduction in NaR cell number in mutants at 4 dpf (quantified in C′,D′), with partial recovery by 7 dpf (unpaired *t*-tests; *trpv6*: 4 dpf: *P*<0.000001, d.f.=20; 7 dpf: *P*=0.0006, d.f.=17; *igfbp5a:* 4 dpf: *P*=0.0001, d.f.=23; 7 dpf: *P*<0.0001, d.f.=17). In B,C′,D′, dashed lines indicate the median. ns, not significant; WT, wild type. Scale bars: 100 µm.

Calcium is taken up from the environment in fish larvae through Trpv6 channels present on the surface of specialized NaR ionocytes in the skin (Dymowska et al., 2012). NaR cells uniquely express *igfbp5a* (Dai et al., 2010, 2014) and represent a subset of ionocytes expressing Na^+^/K^+^ ATPase (Hwang and Chou, 2013). Immunostaining for Na^+^/K^+^ ATPase combined with the *SOX10:*Cre lineage label (driven by a human neural crest-specific enhancer) in otherwise wild-type fish confirmed that ionocytes (including the NaR subset) do not derive from neural crest (Fig. 4A), but rather are ectodermal (Janicke et al., 2007). We then questioned whether the persistently low calcium content of *sox10* mutants could be due to a deficiency of *trpv6* expression or a reduction in the number of NaR ionocytes. Although rt-PCR revealed no overt change in whole-body *trpv6* levels (Fig. 4B), possibly due to low-level expression elsewhere in the body (Lin et al., 2011), we did detect significant decreases in the numbers of *trpv6^+^* and *igfbp5a*^+^ cells at 4 dpf, with mild recovery by 7 dpf (Fig. 4C-D′). These patterns support reduced NaR number as the cause of the systemic calcium deficit and the associated lack of bone mineralization. Whether per-NaR cell *trpv6* transcription is also affected remains to be determined. Published single-cell RNAseq data confirm that differentiating NaR cells contain no *sox10* transcripts (Sur et al., 2023). The NaR deficit in *sox10* mutants therefore cannot be explained by a simple cell-autonomous requirement for Sox10.

NaR cell numbers fluctuate depending on the amount of calcium in the environment, with low Ca^2+^ stimulating their proliferation and thereby increasing Ca^2+^ uptake, versus minimal proliferation and uptake under high Ca^2+^ conditions (Kwong et al., 2014; Lin and Hwang, 2016). These fluctuations were also observed in *sox10* mutants (Fig. S5A-D), indicating that they are still capable of responding to environmental conditions. However, the increase in NaR cells observed in mutants raised at low Ca^2+^ is dampened relative to controls, apparently insufficient to raise total Ca^2+^ content (Fig. S4A) or permit robust skeletal mineralization (Fig. S4B).

### Endocrine suppression of NaR ionocyte expansion in *sox10* mutants

**Fig. 5.**
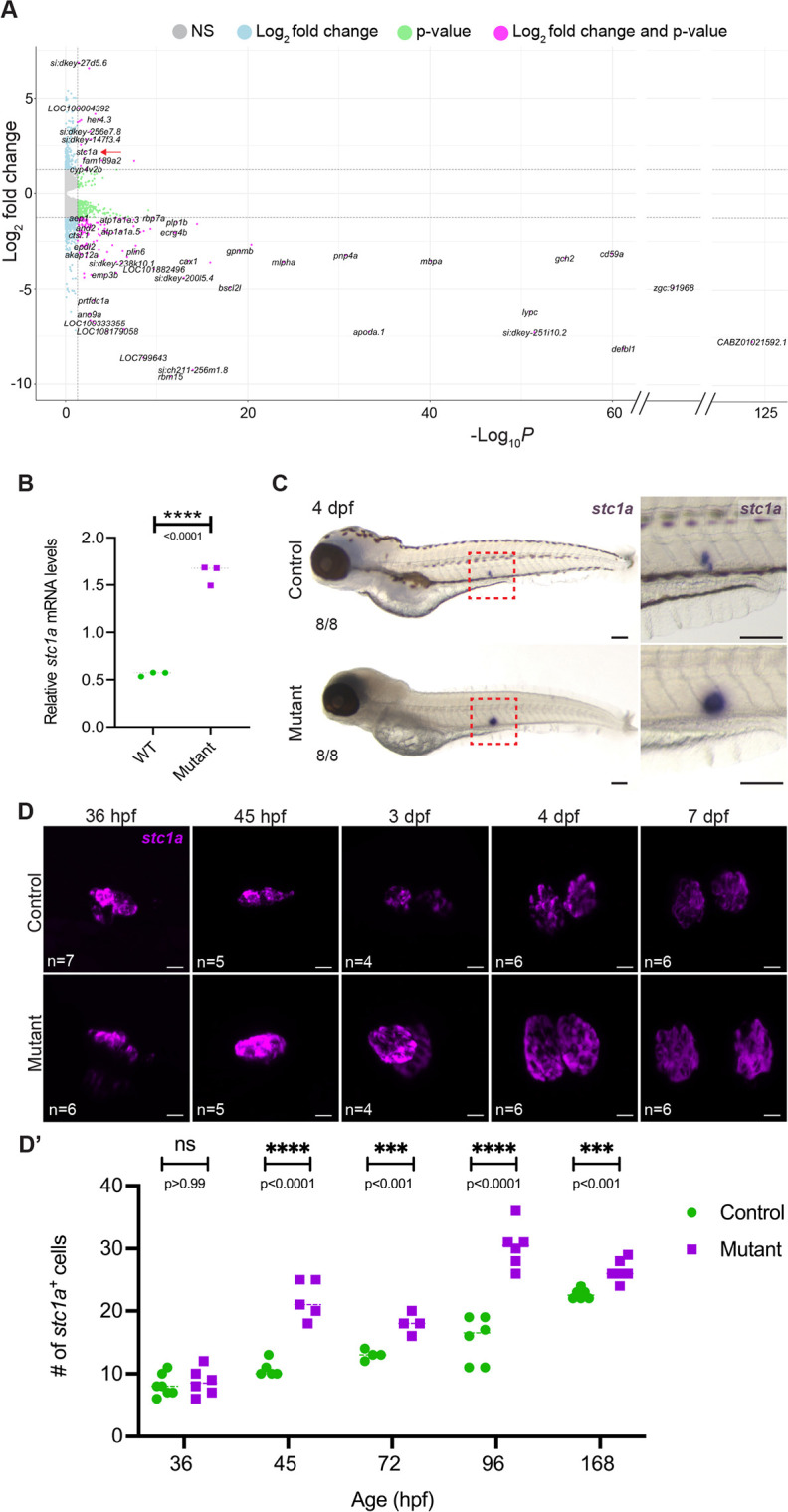
**Upregulation of anti-hypercalcemic hormone *stc1a* in *sox10* mutants.** (A) Volcano plot of whole-body bulk RNAseq shows *stc1a* as significantly upregulated in the *sox10* mutants at 4 dpf (*P*-value cutoff: 0.05; fold change cutoff: 1.25). Red arrow points to *stc1a.* (B,C) Both semi-quantitative rt-PCR (B) and *in situ* hybridization (C) detect a robust upregulation of *stc1a* mRNA in *sox10* mutants at 4 dpf (unpaired *t*-test; *P*<0.0001, d.f.=4). Red boxes in C indicate the regions shown at higher magnification on the right. Numbers in panels indicate the proportion of larvae of that genotype with the presented phenotype. Scale bars: 100 µm. (D,D′) The increase in *stc1a* transcript levels is due at least in part to an increase in the number of *stc1a^+^* cells in *sox10* mutant corpuscles, first detected at 45 hpf and resolving at 7 dpf (unpaired *t*-tests; 36 hpf: *P*=0.640, d.f.=11; 45 hpf: *P*=0.00009, d.f.=8; 72 hpf: *P*=0.002, d.f.=6; 96 hpf: *P*=0.00003, d.f.=10; 168 hpf: *P*=0.0007, d.f.=10. ns, not significant; WT, wild type. Scale bars in D: 10 µm.

**Fig. 6.**
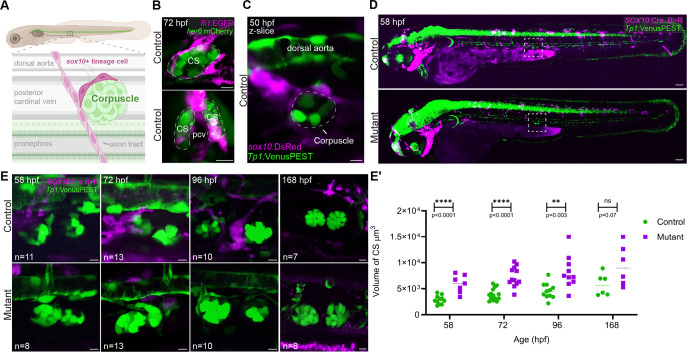
***sox10*^+^ crest-derived cells surround the CS and are missing in *sox10* mutants.** (A) Schematic of the corpuscles' position and surrounding environs. The glands are positioned ventral to the dorsal aorta, dorsal to the pronephros, and flanking the posterior cardinal vein (pcv). *sox10^+^* lineage cells branch off neighboring axon tracts (lined with sox10^+^ Schwann cells and Schwann cell precursors) to contact the corpuscles. (B) Control 72 hpf larva doubly transgenic for *fli1:*EGFP (magenta; labeling vasculature) and *her6:*mCherry (green; labeling the corpuscles). Top image is a single optical section from the lateral perspective showing endothelial cells wrapping around the CS. Bottom image is a maximum intensity projection rotated orthogonally to show the close interaction of the CS with the pcv. (C) Single optical section showing *sox10:*DsRed^+^ cells in close contact with the *Tp1*:VenusPEST^+^ corpuscles (outlined) by 50 hpf. (D,E) Whole-mount live imaging of *SOX10:Cre B>R; Tp1*:VenusPEST fish showing *sox10*^+^ lineage cells surrounding the CS at earlier stages (58 and 72 hpf), nearby at 96 and 168 hpf, and absent in the *sox10* mutants. The entire fish is shown in D to highlight the deficiency of trunk NCCs (magenta) with the box indicating the region shown in E. (E′) Volume measurements of control and mutant *Tp1:*VenusPEST^+^ CS between 58 and 168 hpf. Only the right CS, closest to the microscope lens, was measured. (unpaired *t*-tests; 58 hpf: *P*=0.0001, d.f.=16; 72 hpf: *P*=0.00001, d.f.=24; 96 hpf: *P*=0.003, d.f.=19; 168 hpf: *P*=0.068, d.f.=10). In E′, dashed lines indicate the median. ns, not significant. Scale bars: 10 µm (B,D,E); 100 µm (C).

The fact that the number of *trpv6*^+^ NaR cells remains low in *sox10* mutants despite their clear need for calcium struck us as paradoxical. We reasoned that mutants might be lacking a factor that stimulates NaR proliferation, or, conversely, have too much of a different factor that blocks their increase. Our whole-body RNAseq analysis at 4 dpf revealed stanniocalcin 1 (*stc1a*) as one of the few genes significantly upregulated in *sox10* mutants and the only one directly involved in calcium homeostasis (Fig. 5A; Tables S1, S2). We confirmed this by rt-PCR, demonstrating a threefold upregulation of *stc1a* in mutants (Fig. 5B). Stc1a is an anti-hypercalcemic hormone triggered by high environmental calcium through activation of the Calcium-Sensing Receptor (CaSR) (Radman et al., 2002; Tseng et al., 2009; Lin et al., 2014, 2017). Stc1a reduces calcium uptake to maintain physiologically safe levels by inhibiting proliferation of NaR cells and suppressing *trpv6* expression (Liu et al., 2020; Li et al., 2021). The dominant sources of Stc1a in fish larvae are the CS, teleost-specific glands that bud off the distal pronephros by 50 hpf and are positioned to either side of the posterior cardinal vein with their own vascular supply by 3 dpf (Fig. 6A,B) (Wendelaar Bonga et al., 1977; Cheng and Wingert, 2015; Naylor et al., 2018). *stc1a* expression is detectable prior to full CS extrusion (Naylor et al., 2018) and is thus potentially involved in maintaining calcium balance as early as 24 hpf.

Aberrantly elevated *stc1a* expression in *sox10* mutants might thus explain their reduced number of NaR cells and calcium uptake. *In situ* analyses showed that the robust increase first becomes apparent after completion of CS extrusion (after 36 hpf; Fig. 5D), is most obvious at 4 dpf (Fig. 5C,D), then begins to level out by 7 dpf (Fig. 5D), when both *trpv6^+^* NaR cell numbers and mineralization are partially recovering. The *stc1a* increase is due at least in part to higher numbers of *stc1a^+^* cells in the mutant CS between 45 hpf and 4 dpf (*P*<0.001, unpaired *t*-test; Fig. 5D′). Interestingly, in low-Ca^2+^ medium, *stc1a* expression was undetectable in controls but merely reduced in mutants (Fig. S5E), possibly explaining why mutants still have fewer NaR cells and less calcium uptake than their siblings under these conditions (Figs S4A, S5A).

The *stc1a*-expressing corpuscles are derived from intermediate mesoderm (Naylor et al., 2016, 2018) and never pass through a *sox10^+^* state, so their dysfunction in *sox10* mutants must also be indirect. We looked for *sox10* lineage^+^ cells in or surrounding the glands, predicting that they may be aberrant or missing in mutants. We tracked neural crest using *SOX10*:Cre (Kague et al., 2012) in combination with the *actb2:*BFP*>*DsRed Cre reporter (Kobayashi et al., 2014) and all recently *sox10*-expressing cells using *sox10*:DsRed (driven by the 4.9-kb zebrafish *sox10* promoter; Das and Crump, 2012). All traces were performed in combination with the *Tp1:*VenusPEST Notch reporter (Ninov et al., 2012) or the *her6:*mCherry reporter (Kraus et al., 2022), both of which are expressed in the CS after ∼36 hpf. We detected close physical interactions between the CS and *sox10:*DsRed*^+^* or *SOX10:*Cre-traced cells at 50 and 72 hpf (Fig. 6C), after the glands had fully formed. Direct contact was observed in 47% (8/17) and 65% (9/14) of DsRed^+^ and Cre^+^ embryos, respectively, at 50 hpf (see Movie 1), but only in ∼25% (5/19) at 72 hpf. Lineage-traced crest remained in the general vicinity of the CS up to 7 dpf but were much less likely to be captured in close contact (Fig. 6D,E), suggesting the interactions may be transient and/or dynamic. Strikingly, *sox10* mutants lacked *SOX10*:Cre lineage-labeled cells around the CS at all stages examined (Fig. 6D,E). This is consistent with the complete or near-complete loss of many neural crest cell sublineages previously reported in *sox10* mutant models (Kelsh and Eisen, 2000; Dutton et al., 2001; Carney et al., 2006). Mutant VenusPEST^+^ CS cells were less organized, and mutant gland volume was larger (*P*<0.0001 at 58 and 72 hpf; *P*=0.003 at 96 hpf; not significant at 168 hpf; unpaired *t*-tests; Fig. 6E′). These patterns suggest that *sox10^+^* crest-derived cells may act locally to restrain CS growth and *stc1a* expression to regulate embryonic calcium homeostasis.

### *stc1a* is epistatic to *sox10* and the proximate cause of the mineralization deficit

**Fig. 7.**
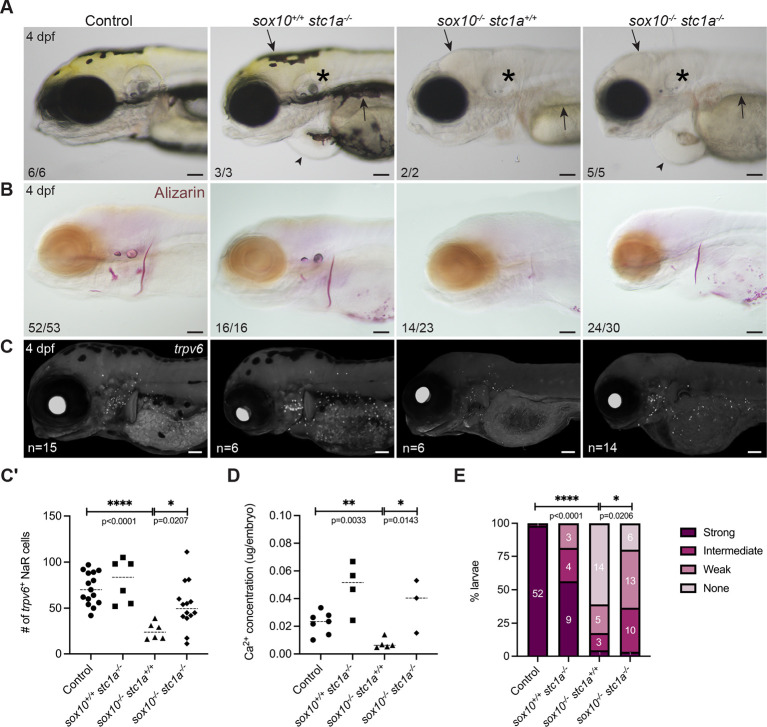
***stc1a* is epistatic to *sox10* in control of systemic calcium content.** (A) Brightfield images of *sox10* and *stc1a* controls and mutants at 4 dpf. Double mutants phenocopy the loss of pigment (arrows) and the inner ear malformations (asterisks) of single *sox10* mutants and the cardiac edema of the *stc1a* mutant (arrowheads). (B-C′) Loss of *stc1a* on the *sox10* mutant background improves mineralization (B) and the number of *trpv6^+^* ionocytes (C) at 4 dpf, quantified in C′ (unpaired *t*-test; *P*=0.0207, d.f.=18). In A,B, numbers in panels indicate the proportion of larvae of that genotype with the presented phenotype. (D) Calcium quantification shows an increase (unpaired *t*-test; *P*=0.143, d.f.=6) in calcium levels in *sox10^−/−^; stc1a^−/−^* compared to *sox10^−/−^.* (E) Quantitation of mineralization levels in *sox10; stc1a* clutches grouped based on the intensity of Alizarin Red staining. There was a significant increase in the proportion of double mutants with detectable mineralization compared with *sox10* single mutants (χ^2^; *P*=0.0206, d.f.=3). In C′-E, ‘control’ includes wild-type and heterozygous larvae. Dashed lines indicate the median. Scale bars: 100 µm.

Our results thus far suggested that the absence of *sox10*^+^ cells leads to unrestrained growth and Stc1a production by the corpuscles, in turn inhibiting NaR cell proliferation and preventing sufficient calcium uptake for mineralization. To test this model, we performed an epistasis assay of *stc1a* on the *sox10* mutant background using the previously reported *stc1a^mi610^* mutant (Li et al., 2021). *sox10^ci3020^; stc1a^mi610^* double mutants present both the trademark lack of pigmentation and underdeveloped inner ears of *sox10* single mutants alongside the characteristic cardiac edema of *stc1a* mutants (Fig. 7A), supporting that these phenotypes are genetically independent. However, bone mineralization was strikingly improved in double mutants relative to *sox10* single mutants at 4 dpf (Fig. 7B). Eighty percent of the double mutants (24/30) exhibited Alizarin Red staining: 13 weakly, ten at an intermediate level, and one strongly (Fig. 7E; also see Fig. S6B for examples). It is worth noting that the presence of cardiac edema in the double mutants may have compromised bone formation in some individuals. For comparison, among 23 *sox10^−/−^; stc1a^+/+^* clutchmates, 14 had no staining, five had weak staining, three intermediate, and one strong (Fig. S6B; *P*=0.0206, χ^2^ test). In the original *sox10^ci3020^* single mutant crosses, only three out of 48 single mutants showed intermediate or weak staining; the other 45 had none (Fig. S6A), suggesting the presence of genetic modifiers. We also noted significant improvement in NaR cell number and calcium content in the double *sox10; stc1a* mutants relative to *sox10* single mutants (Fig. 7C′,D), further supporting that *stc1a* is epistatic to *sox10* in mineral regulation.

## DISCUSSION

### Sox10 requirement in bone mineralization

This study reveals a previously undescribed, indirect role for Sox10 in skeletal mineralization. Two independent *sox10* mutant lines exhibit delayed and reduced mineralization of all bones, no matter their embryonic origin or ossification type. Mutant osteoblasts appear to differentiate normally (Fig. 2D) and gradually lay down ECM to create typically sized bone templates (Fig. 2C-E). However, their transcriptomes may be subtly altered: we detected changes in whole-body mRNA levels of genes encoding osteoblast-enriched enzymes involved in regulation of phosphate availability and homeostasis (*phospho1*, *alpl*, *enpp1*, *entpd5*) or bone accessory proteins (*spp1* (osteopontin) (Fig. S2). We posit that these shifts may reflect secondary transcriptional responses of osteoblasts to the major systemic calcium deficit or altered inorganic phosphate availability incurred by calcium depletion. Reduced levels of certain factors may exacerbate the mineralization defect, as other studies have demonstrated that partial or complete genetic loss of some of these accessory proteins and enzymes can decrease bone mineral density and/or impair mineralization (Yadav et al., 2011; Huitema et al., 2012a; Christov and Juppner, 2013; Si et al., 2020).

We noted with interest the changes in phosphate regulators, given that we did not measure any consistent differences in mutants' total phosphate content by a colorimetric assay (Fig. 4C). It is possible that the assay is insufficiently sensitive or overwhelmed by maternally deposited yolk stores (Miyares et al., 2014; Fraher et al., 2016; Quinlivan and Farber, 2017). However, how osteoblast-produced inorganic phosphate intended for hydroxyapatite formation is managed when calcium is not available is an intriguing question. Of note, in our comparison of bone stains, we observed recovery of Von Kossa staining before that of Alizarin Red, calcein, or OsteoImage (hydroxyapatite). In Von Kossa staining, silver cations from the silver nitrate staining solution interact with calcium phosphate to produce a yellowish silver phosphate, which subsequently blackens surrounding organic matter (Puchtler and Meloan, 1978; Meloan and Puchtler, 1985; Santos et al., 2023). It is possible that the early recovery of this stain reflects a reaction with inorganic phosphate that accumulates in the bone matrix due to the calcium deficit.

Rearing *sox10* mutant embryos in a high-Ca^2+^ medium did not improve their mineralization deficit, in contrast to prior results for similarly under-mineralized *trpv6* and *msp* mutants (Vanoevelen et al., 2011; Huitema et al., 2012b). A possible explanation for this discrepancy lies in their associated NaR cell phenotypes: *trpv6* mutants have excess NaR cells (Xin et al., 2019), compared with the depletion observed in *sox10* mutants (Fig. 4). Although Trpv6 is the only route of free calcium ingress in ionocytes, NaR cells express two other transporters capable of moving calcium across membranes: the Na^+^/Ca^2+^ exchanger (also known as Ncx1b, encoded by *slc8a1b*) and the plasma membrane Ca^2+^-ATPase (also known as Pmca2, encoded by *atp2b2*) (Liao et al., 2007). The Na^+^/Ca^2+^ exchanger normally removes calcium from cells but can also work in the reverse direction depending on ion gradients (Yu and Choi, 1997). Given enough excess NaR cells and very high external Ca^2+^ (as would be the case for *trpv6* mutants raised in high Ca^2+^; Vanoevelen et al., 2011), it is conceivable that Ncx1b might import sufficient Ca^2+^ to initiate mineralization. This mechanism would not be available in *sox10* mutants, which have almost no NaR cells at high Ca^2+^ (Fig. S5A).

How calcium uptake and bone mineralization begin to recover in *sox10* mutants is still an open question. One possibility is that other endocrine hormones gradually counteract elevated Stc1a activity. Parathyroid hormone and vitamin D, both reported to have hypercalcemic properties in fish as well as in mammals, act to increase Trpv6-mediated calcium uptake (Lin et al., 2012, 2022; Khundmiri et al., 2016). Zebrafish lack parathyroid glands, but express parathyroid hormones in the central nervous system and sensory neuromasts (Hogan et al., 2005). Similarly, fish synthesize vitamin D as early as 3 dpf in response to decreased environmental calcium (Lin et al., 2022). Other physiological changes are occurring at the same time as mineralization recovery begins, including maturation of the digestive tract and auxiliary endodermal organs (Guerrera et al., 2015) and yolk depletion (Quinlivan and Farber, 2017). Although *sox10* mutants lack an enteric nervous system (Kelsh and Eisen, 2000) and are not fed in our experiments, it is possible that passage of embryo medium through the digestive tract allows calcium uptake through intestinal enterocytes, contributing to the mutants' partial recovery. We have also observed ectopic calcium/hydroxyapatite deposits in the yolk area of mutants at 3 and 4 dpf that begin to resolve coincident with the onset of bone mineralization (Fig. 1C,D). The calcium in those deposits could conceivably be remobilized and made available for forming bones as the yolk is depleted. Two other zebrafish mutants that lack mineralization during larval stages (*msp* (Huitema et al., 2012b) and *her9* (Stenzel et al., 2022)] also naturally recover to some extent, supporting robustness or complementarity in mechanisms driving calcium uptake for skeletal mineralization.

### Sox10 drives bone mineralization indirectly through interactions with endocrine glands involved in calcium homeostasis

The most striking finding from the whole-body transcriptional analysis was the tripled *stc1a* mRNA levels in *sox10* mutants (Fig. 5A,B). High Stc1a blocks proliferation of *trpv6^+^* ionocytes (Li et al., 2021), reducing calcium uptake. That elevated *stc1a* is the major driver of the calcium deficit in *sox10* mutants was confirmed by our epistasis studies (Fig. 7). However, whether the increase in *stc1a* mRNA is attributable solely to higher cell number in the mutant corpuscles (Fig. 5D,D′) or also to a per-cell increase in transcription is not yet clear. The cells themselves may also be larger (compare CS cell size at 168 hpf in Fig. 6E). Previous studies have shown that high external calcium upregulates *stc1a* transcription at least in part via CaSR, which is also expressed in the CS (Lin et al., 2017). Aberrant activity of CaSR in the absence of *sox10*^+^ lineage cells could therefore potentially boost *stc1a* transcription. In support of the idea that the *stc1a* increase is more complex than just increased CS cell number, another mineral-regulating hormone enriched in the CS, *fgf23* (Elizondo et al., 2010; Mangos et al., 2012), is downregulated in *sox10* mutants (Fig. S2B) despite the increased size of the corpuscles. Fgf23 has anti-hypercalcemic effects similar to Stc1a, reducing Ca^2+^ uptake in conditions of high systemic calcium, in addition to regulating phosphate homeostasis (Lin et al., 2017; Rodrat et al., 2023; Martinez-Heredia et al., 2024); its low expression in *sox10* mutants is consistent with their calcium deficit (Lin et al., 2017). Published single-cell RNAseq data (Sur et al., 2023) show that corpuscle cells also express receptors for other endocrine factors involved in mineralization between 2 and 4 dpf, including receptors for calcitonin (*calcr*), cortisol (*nr3c1*), vitamin D (*vdrb*), Fgf23 (*fgfr1b*), and Msp (*mst1rb*). It remains to be seen how these pathways are affected in the absence of *sox10*^+^ cells and whether they are involved in *stc1a* upregulation.

Formation of the CS from the pronephros depends on key signaling pathways such as FGF, Wnt, and Notch. However, the regulatory mechanisms that govern gland size after their initial budding remain unclear. Single-cell RNAseq data (Sur et al., 2023) and supporting studies (Drummond et al., 2017; Naylor et al., 2018; Klingbeil et al., 2022) indicate that the CS continue to express components of these signaling pathways at 55-120 hpf, after budding is completed*.* Moreover, live-imaging experiments (Fig. 6) confirm active Notch signaling in fully formed corpuscles, as evidenced by the reporters *Tp1:VenusPEST* and *her6:mCherry*. It is plausible that *sox10^+^* cells communicate with the CS through one or more of these pathways, with disrupted signaling affecting CS cell proliferation and stanniocalcin production in mutants.

The question of why the corpuscles, derived from a *sox10*-negative mesodermal lineage, are profoundly affected by loss of *sox10* is not fully resolved. We did not observe an increase in *stc1a*^+^ cell number before 2 dpf, i.e. only after the glands had fully extruded from the pronephros, ruling out expanded CS specification as the explanation for the larger glands (as previously found in other mutant lines; Cheng and Wingert, 2015; Drummond et al., 2017). Our experiments instead revealed that a *sox10*^+^ sublineage interacts with these glands post-extrusion, and that these neural crest-derived cells are missing in *sox10* mutants (Fig. 6C-E), like many other crest derivatives (Herbarth et al., 1998; Southard-Smith et al., 1998; Kelsh and Eisen, 2000; Dutton et al., 2001). A tantalizing possibility is that they may be precursors of the sympathetic neurons that will innervate the CS in adults (Krishnamurthy and Bern, 1971; Wendelaar Bonga et al., 1977). Sympathetic neurons derive from *sox10*^+^ neural crest, specifically from so-called Schwann cell precursors (SCPs) (Kamenev et al., 2021). Differentiating Schwann cells and SCPs may be the predominant *sox10*^+^ cell types lining the trunk sensory and motor axon tracts that pass by the corpuscles (Kuhlbrodt et al., 1998; Kamenev et al., 2021), from which we see cells emerging to contact the glands directly (Fig. 6C). Schwann cells and SCPs are largely absent in *sox10* mutant fish and mice (Kelsh and Eisen, 2000; Britsch et al., 2001). Interestingly, hallmark signs of sympathetic neuronal differentiation in the trunk are not evident in wild-type zebrafish until around 7 dpf (An et al., 2002), well after this CS phenotype arises. The regulatory interaction between the *sox10*^+^ lineage cells and the CS is thus expected to be non-neuronal in nature at these early stages. Although mutant lethality makes it challenging to study the onset of sympathetic control, we expect that the requirement for *sox10*^+^ lineage cells in managing stanniocalcin production and/or secretion, and thus calcium homeostasis, persists throughout the lifespan.

Humans and other mammals produce stanniocalcin hormones, but lack a gland homologous to the CS (Yeung et al., 2012). If the mineralization deficits in fish *sox10* mutants result solely from dysregulated corpuscle development, mammals with *Sox10* loss may not exhibit similar mineralization issues. However, our studies prompt the broader notion that crest-derived cells destined to become part of the sympathetic nervous system may interact with and begin regulating their target organs' growth and activity earlier in embryonic development than previously appreciated. This could potentially drive physiological and endocrinological symptoms in individuals with congenital neurocristopathies caused by deficient crest production or survival (Vega-Lopez et al., 2018).

## MATERIALS AND METHODS

### Zebrafish husbandry and lines

Zebrafish (*Danio rerio*) embryos were grown at 28.5°C in standard embryo medium (EM) (Westerfield, 2007) unless otherwise noted: 15 mM NaCl, 0.5 mM KCl, 1 mM CaCl_2_·2H_2_O, 0.15 mM KH_2_PO_4_, 0.06 mM NaH_2_PO_4_ and 1 mM MgSO_4_·7H_2_O. Published mutant and transgenic lines used here include *sox10^ci3020^* (Okeke et al., 2022), *sox10^m618^* (Malicki et al., 1996), *stc1l^mi610^* (Li et al., 2021), *Tg(Hsa.RUNX2:mCherry)^zf3244^* (alias *RUNX2:mCherry*) (Barske et al., 2020), *Tg(sp7*:*EGFP)^b1212^* (DeLaurier et al., 2010), *Tg(Ola.Bglap:EGFP)^hu4008^* (alias *osc:EGFP*) (Vanoevelen et al., 2011), *Tg(Mmu.Sox10-Mmu-Fos:Cre)^zf384^* (alias *SOX10*:*Cre*) (Kague et al., 2012), *Tg(EPV.TP1-Mmu.Hbb:Venus-Mmu.Odc1)^s940^* (alias *Tp1:VenusPEST*) (Ninov et al., 2012), *Tg(fli1:EGFP)^y1^* (Lawson and Weinstein, 2002), *Tg(Xla.Eef1a1:loxP-DsRed2-loxP-EGFP)^zf284^* (alias *ef1a:DsRed>EGFP*) (Kague et al., 2012), *Tg(actb2:LOXP-BFP-LOXP-DsRed)^sd27^* (alias *actb2:*BFP*>*DsRed) (Kobayashi et al., 2014) and *Tg(her6:mCherry)^sd64^* (Kraus et al., 2022). Lines were maintained as hetero- or hemizygotes.

### Bone staining

For all fixed bone stains, zebrafish larvae were fully anesthetized with MS-222 (also known as Tricaine, Syndel) at the desired stage and then fixed in 2% paraformaldehyde (250 µl EM, 250 µl 4% paraformaldehyde, and 500 µl PBS with 0.1% Tween) overnight at 4°C or for 1 h at room temperature. For Alizarin Red staining alone, following fixation larvae were rinsed twice in 25% glycerol in 0.5% KOH for 10 min each and stained with 0.01% Alizarin in 25% glycerol/100 mM Tris pH 7.5 for 4 h at room temperature. They were then bleached for 10 min in 3% H_2_O_2_ in 0.5% KOH under a light source. Specimens were stored and imaged in 50% glycerol in 0.5% KOH or 100% glycerol immediately to prevent fading (adapted from Jiang et al., 2020). Combined Alcian Blue and Alizarin Red staining was performed as described previously (Walker and Kimmel, 2007). For Von Kossa staining, fixed embryos were rinsed with deionized water and stained with 2.5% silver nitrate solution (Abcam, ab150687) under a light source for 20 min. The reaction was stopped with 5% sodium thiosulfate to prevent overstaining, and larvae were imaged immediately (Paese et al., 2022; Khrystoforova et al., 2022). For the OsteoImage™ Mineralization Assay (Lonza Bioscience, PA-1503), we followed the manufacturer's protocol after fixation. Briefly, fixed larvae were rinsed with diluted wash buffer then stained in diluted staining reagent for 30 min at room temperature in the dark. Before imaging, they were rinsed three times with wash buffer for 5 min each. For live staining, larvae were incubated in Alizarin Red (0.03 mg/ml in 30 ml EM) for 2 h at 28.5°C or in Calcein Green (0.1 mg/ml in 30 ml EM) at 28.5°C overnight (Teng et al., 2018). For each round of each bone-staining experiment, a minimum of six individuals were stained and imaged per genotype/stage/group.

### Calcium and phosphate supplementation and depletion treatments

For calcium treatments, the amount of CaCl_2_·2H_2_O was increased two- or tenfold for 2 mM and 10 mM treatments, respectively, completely removed (0 mM), or decreased to 0.02 mM (Huitema et al., 2012b). For the high phosphate treatment (adapted from Huitema et al., 2012a), the concentrations of KH_2_PO_4_ and NaH_2_PO_4_ were raised to 0.5 mM and 9.5 mM, respectively, to increase the total PO_4_^3−^ to 10 mM, thereby maintaining the proportional K^+^/Na^+^ ratio as in the control EM. The ‘No PO_4_^3−^’ treatment included neither KH_2_PO_4_ nor NaH_2_PO_4_ in the media. Embryos were placed into the different treatment media at 4 hpf and kept there until 4 dpf. A stock of each treatment media was prepared on the first day, and the medium in each dish was replaced daily. A minimum of six control and six mutant larvae were used per treatment group, and all treatments were repeated at least twice.

### Thyroid hormone treatment

T3 (T2877, Sigma-Aldrich) was added to embryo media at approximately 32 hpf, coinciding with the onset of thyroid gland activity. T3 was administered at concentrations of 50, 100, 300, and 600 µgl^−1^ in 30 ml of embryo media, following established protocols (Zada et al., 2016; Walter et al., 2019). The media was refreshed daily until 4 dpf, after which skeletal staining was performed to assess mineralization. Each treatment was conducted in four independent experiments, with at least six *sox10* mutants and six wild-type sibling controls included per group. This ensured sufficient replicates to evaluate the effects of T3 on skeletal development.

### Whole-mount *in situ* hybridization and immunostaining

cDNAs for *stc1a*, *trpv6*, *igfbp5a*, *col10a1a*, *phospho1*, *sparc*, *spp1*, *runx2a*, and *runx2b* (Paul et al., 2016) were amplified with Herculase II Fusion DNA Polymerase (Agilent) (see Table S3 for primer sequences) and inserted into the pCR-Blunt II-TOPO vector (Thermo Fisher Scientific). After sequence confirmation and linearization by restriction digest, antisense probes were synthesized from each plasmid using Sp6 or T7 polymerase and digoxigenin-tagged nucleotides (Roche). Colorimetric and fluorescence *in situ* hybridizations were performed as described previously (Barske et al., 2018). Colorimetric *in situ* signals were developed with either NBT-BCIP or BM Purple (Sigma-Aldrich), whereas fluorescence *in situ* signals were developed with TSA Cyanine 3 (Akoya Biosciences). Immunostaining was performed as described (Okeke et al., 2022). Primary antibodies used were anti-Na^+^/K^+^ ATPase (1:400; Developmental Studies Hybridoma Bank, a5, RRID:AB_2166869) and anti-Sox10 (1:500; Genetex, GTX128374, RRID:AB_2885766), used with Alexa Fluor 647- and 568- conjugated goat anti-mouse and goat anti-rabbit secondary antibodies (1:250; Thermo Fisher Scientific; A32728, RRID:AB_2633277; A11011, RRID:AB_143157). In both procedures, permeabilization steps were skipped for markers limited to surface expression (*trpv6* and *igfbp5a in situ*, and a5 immunostaining). A minimum of six control and six mutant larvae were stained and imaged for each marker, and the experiments were repeated at least twice.

### Bromodeoxyuridine staining

Control and mutant larvae were incubated in 15 mM 5-bromo-2′-deoxyuridine (Sigma-Aldrich, B5002) for 30 min at 28.5°C in the dark. Following incubation, they were rinsed with embryo media, fixed for 2 h at room temperature, and stored in 100% methanol at −80°C until further processing. The larvae were rehydrated by sequentially walking them out of methanol, permeabilized using proteinase K, post-fixed, and rinsed thoroughly. They were then treated with 2 N HCl for 1 h at room temperature to denature their DNA. Sequential immunostaining was performed, incubating first with the primary a5 antibody (1:400 as above), followed by the 647-conjugated goat-anti-mouse secondary antibody, then with a primary anti-BrdU antibody [1:200; BU1/75 (ICR1), Abcam, ab6326, RRID:AB_305426], and a 488-conjugated goat anti-rat secondary antibody (1:250; Thermo Fisher Scientific, A11006, RRID: AB_2534074).

### rt-PCR

rt-PCRs were performed to estimate transcript levels of mineralization-associated genes in *sox10^ci3020^* mutants. Each sample consisted of 10-15 mutant and 10-15 stage-matched wild-type controls that were pooled at 4 dpf and frozen at −80°C. RNA was extracted using the RNAqueous-4PCR Total RNA Isolation Kit (Invitrogen), and equivalent amounts (500 ng) were used to synthesize cDNA with the High-Capacity cDNA Reverse Transcription Kit (Applied Biosystems). rt-PCR was run with a minimum of three biological replicates per genotype, and *eef1g* expression was used for normalization (following Okeke et al., 2022). Band intensity was quantified with Image Lab (Bio-Rad) and analyzed with Prism 10 (GraphPad). Primers, product sizes, and cycling conditions for each gene are listed in Table S4.

### Quantification of mineral content

Whole-body Ca^2+^ and PO_4_^3−^ contents were quantified using colorimetric assay kits (Abcam, ab102505 and ab65622). For Ca^2+^ measurements, 10-15 larvae were pooled at the desired stage in an Eppendorf tube without any liquid, dehydrated at 60°C for 1 h, then digested for at least 4 h in 125 µl of freshly prepared 1 M HCl in an Eppendorf Thermomixer set at 95°C and 750 rpm. The samples were then centrifuged at 4°C for 45 min at 15,000 rpm (21,130 ***g***). Supernatants were distributed on a clear 96-well polystyrene flat-bottomed plate alongside the standard curve reagents prepared according to the protocol provided with the kits. Absorbances were measured on a SpectraMax M5 plate reader. The same procedure was followed for PO_4_^3−^ quantification, with the modification that the supernatants were diluted in deionized water to avoid precipitation.

### Imaging and image analysis

Skeletal stains, brightfield images and colorimetric *in situ* hybridizations were imaged on Zeiss SteREO Discovery.V8 (1.5× objective and 8× magnification) or Zeiss Axioimager.Z1 (10× objective) microscopes, whereas fluorescence *in situ* hybridizations, fluorescent bone stains, live transgenic fish, and immunostained specimens were imaged on a Nikon C2 confocal microscope. High-magnification confocal images were captured with a 40× objective and 2.5× scanning zoom, while the lower magnification whole-body and ventral head pictures were taken with a 10× or 20× objective. Images of red fluorophores and fluorescent proteins were pseudocolored magenta for easier interpretation by people with color blindness. Timelapse imaging of the *SOX10:Cre*; *actb2:*BFP*>*DsRed larva was performed from 48-96 hpf on the Nikon C2 confocal, with an interval of 25 min and at 20× magnification. *trpv6^+^/igfbp5a^+^* ionocytes and *stc1a^+^* cells were quantified using the ‘spots’ option in Imaris 10.1.1. We did not distinguish between gill and skin NaR cells when counting but note that the ventral images (Fig. 4) include predominantly gill ionocytes, while the lateral images (Fig. 7) include both. Lateral images were necessary for the epistasis analysis because the cardiac edema of the double mutants and *stc1a* single mutants prevented the capture of quality ventral images. In the ventral images, we counted cells between the eyes and the cleithrum; in the lateral images, we counted all ionocytes visible on the left side of the larvae, including those in the skin, gills, and over the yolk. CS volumes were measured with the ‘surface labeling’ option in Imaris 10.1.1. A minimum of six replicates were counted for each genotype/stage combination.

### Bulk RNAseq

Ten to 15 mutants and 10-15 stage-matched wild-type sibling control cousins were pooled at 4 dpf and frozen at −80°C. RNA was extracted using the RNAqueous-4PCR Total RNA Isolation Kit (Invitrogen). A minimum of 10 ng µl^−1^ RNA was sent to the Cincinnati Children's Hospital Genomics Sequencing Facility for bulk RNAseq (paired end, 150 bases, 50 million reads per sample; Illumina NovaSeq™ 6000). Raw fastq files were trimmed and subjected to Kallisto for counts (transcripts per million) using the Galaxy platform (Bolger et al., 2014; Bray et al., 2016). Pseudo-aligned reads were then subjected to DESeq2 followed using R Studio (Love et al., 2014). The volcano plot was generated with EnhancedVolcano (Blighe et al., 2018).

### Data analysis

Data analysis was performed with GraphPad Prism (version 10.2.3). *P*-values were calculated with χ^2^ tests or unpaired, two-tailed *t*-tests as noted in the figure legends.

## Supplementary Material



10.1242/develop.204357_sup1 Supplementary information

Table S1. DESeq2 output of sox10 control versus mutant RNAseq analysis
