# Supplementary material for: Sox10 is required for systemic initiation of bone mineralization
Source: Development. 2025 Jan 20;152(2):dev204357. doi: 10.1242/dev.204357 (PMC11833171; doi:10.1242/dev.204357)
Supplement: Supplementary information [file develop-152-204357-s1.pdf]

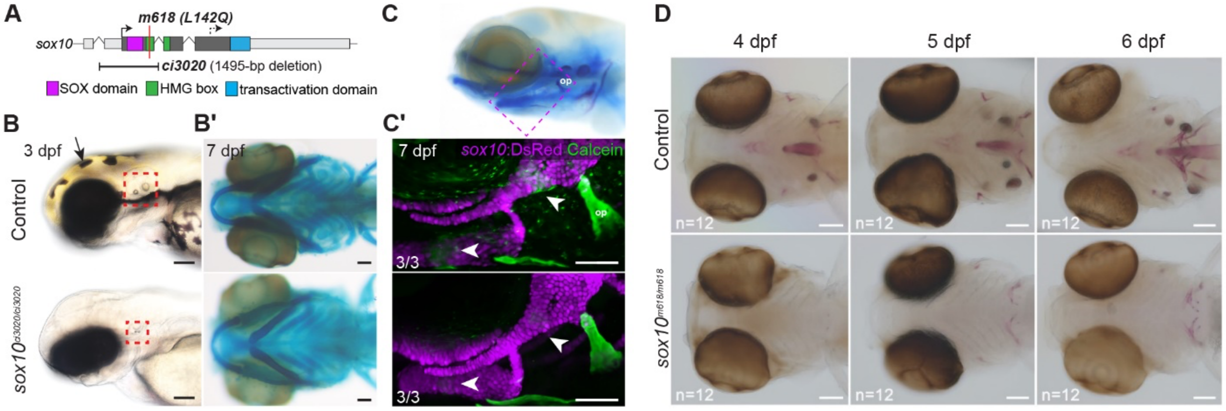

Fig. S1. Validation of *sox10* null allele. (A) Schematic representation of the *ci3020* and *m618* mutations in the *sox10* gene body. Dotted arrow indicates the next in-frame methionine downstream of the *ci3020* deletion. **(B-B')** *sox10*$^{ci3020/ci3020}$ mutants lack melanocytes (arrow) and yellow xanthophores and have inner ear and otolith malformations (dashed red box) (B) but form a normal cartilaginous craniofacial skeleton (Alcian blue staining) (B'). Scale bars: 100 µm. **(C-C')** Lateral view of control embryo stained with Alcian blue and Alizarin red, showing the position of image in C'. *sox10*$^{ci3020/ci3020}$ mutants still show deficient endochondral mineralization at 7 dpf, while intramembranous bones (e.g. op) have begun to catch up. Arrowheads indicate sites of bone collars on the hyomandibular and ceratohyal cartilages. *sox10*:DsRed (magenta) marks chondrocytes, and Calcein green labels mineralized bone. Scale bar: 100 µm. **(D)** The missense *sox10*$^{m618/m618}$ mutant also shows deficient mineralization at 4-6 dpf by Alizarin red staining. Scale bars: 100 µm.

Development • Supplementary information

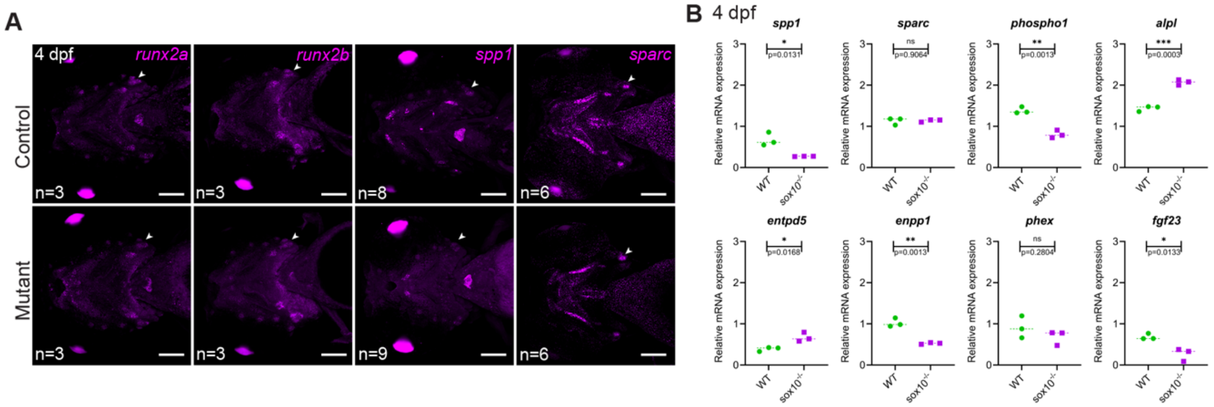

Fig. S2. Altered expression of genes encoding mineralization enzymes and matrix proteins in *sox10* mutants. (A) Fluorescent in situ hybridizations show no changes in *runx2a* or *runx2b* but reduced *spp1* and *sparc* expression. These phenotypes were consistent across all samples imaged. Arrowheads pointing at op. Scale bars: 100 µm. **(B)** Semi-quantitative rt-PCRs were performed on cDNAs made from three pools of 10-15 wild-type (WT) or mutant embryos collected at 4 dpf. The analysis revealed significant increases in *alpl* and *entpd5* (unpaired t-tests: p=0.0003, and p=0.017, respectively, df=4 for both), decreases in *spp1*, *phospho1*, *enpp1*, and *fgf23* (unpaired t-tests: p=0.013, 0.001, 0.001, and 0.013, respectively, df=4 for all), and no changes in *sparc* or *phex* (unpaired t-tests: p=0.906 and p=0.284, respectively, df=4 for both).

Development • Supplementary information

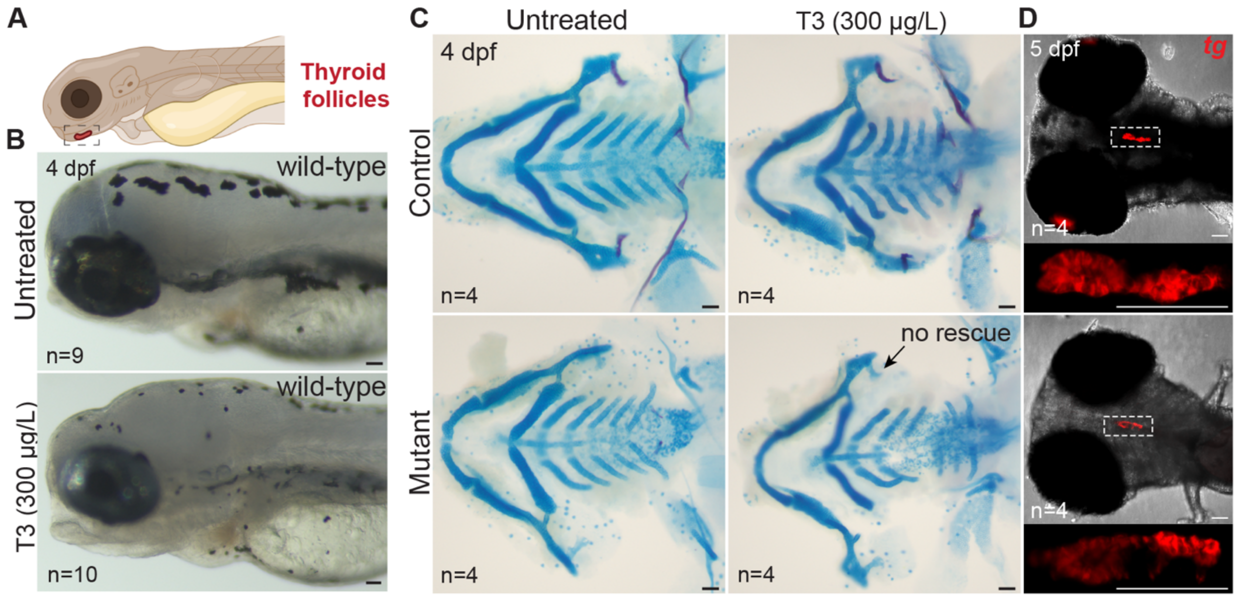

Fig. S3. Normal thyroid gland development and function in *sox10* mutants. (A) Schematic showing location of thyroid follicles in zebrafish larvae. **(B)** Treatment with 300 µgl^{-1} T3 caused reduction in melanocyte coverage in controls at 4 dpf. **(C)** Alcian blue and Alizarin red staining on 4 dpf larvae revealed no rescue of mineralization in *sox10* mutants treated with T3 (arrow points to op bone). **(D)** Fluorescent in situ hybridization demonstrating normal morphology of *tg*+ thyroid follicles in 5 dpf mutants. Scale bars: 50 µm.

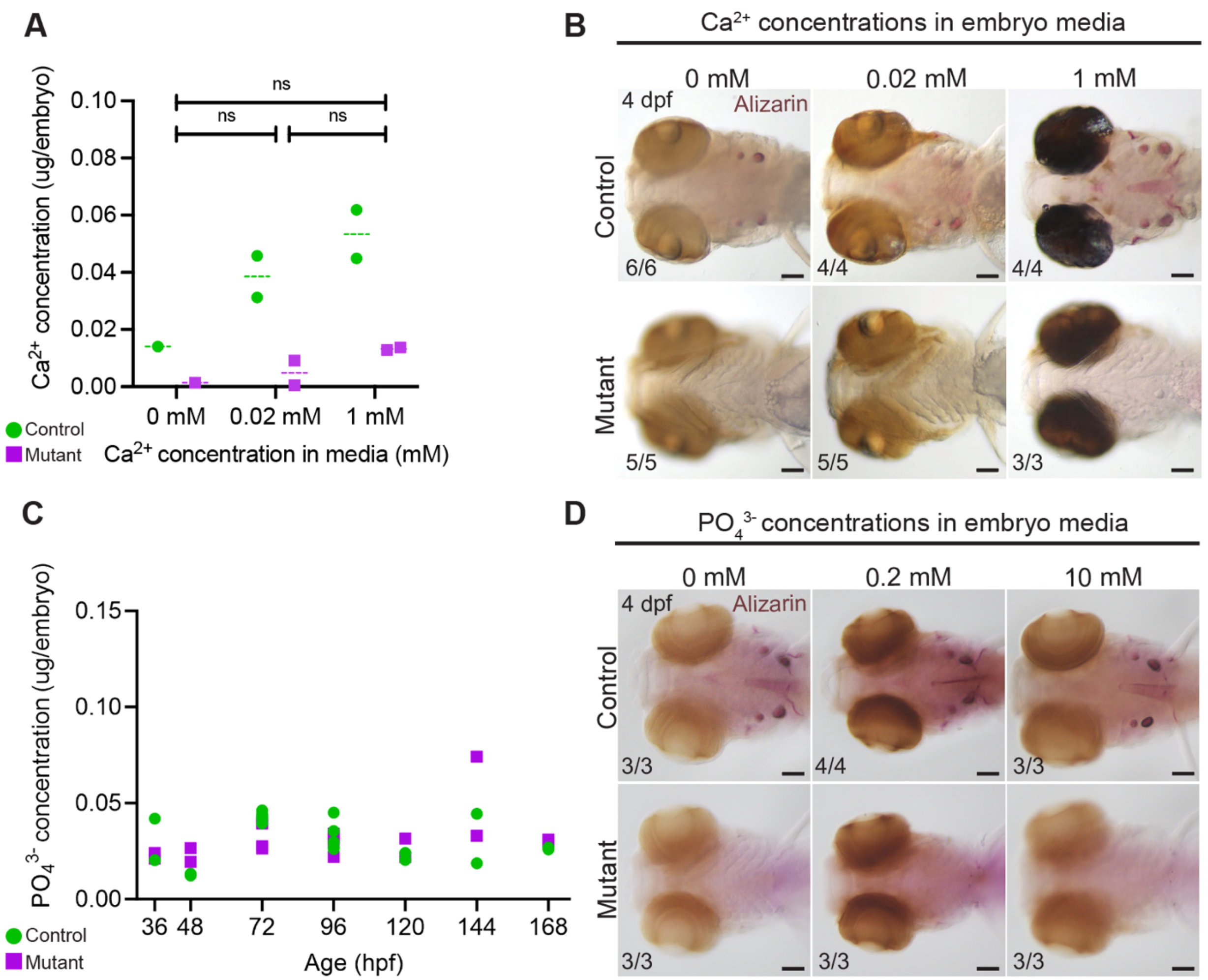

Fig. S4. Neither low calcium nor high or low phosphate improve bone mineralization in *sox10* mutants. (A-B) Calcium levels (A) and Alizarin staining (B) of mutants and controls raised to 4 dpf in different low Ca^{2+} media. Reduction or complete removal of calcium from the embryo media impaired mineralization in control larvae and did not rescue the mutant phenotype (unpaired t-tests: 0.02 vs. 0 mM: p=0.519, df=2; 1 vs. 0 mM: p=0.347, df=2; 1 vs. 0.02 mM: p=0.700, df=2). Otoliths, made from calcium carbonate rather than hydroxyapatite, still form in controls in the absence of external calcium. **(C)** Phosphate levels do not overtly differ between controls and *sox10* mutants throughout larval development (1.5-7 dpf) (unpaired t-tests: 36 hpf: p=0.516, df=2; 48 hpf: p=0.104, df=2; 72 hpf: p=0.046, df=8; 96 hpf: p=0.550, df=8; 120 hpf: p=0.474, df=2; 144 hpf: p=0.462, df=2; 168 hpf: p=0.005, df=4). **(D)** Increasing (10 mM) or removing (0 mM) phosphate from the embryo media does not improve mineralization in *sox10* mutants. Scale bars: 100 µm.

Development • Supplementary information

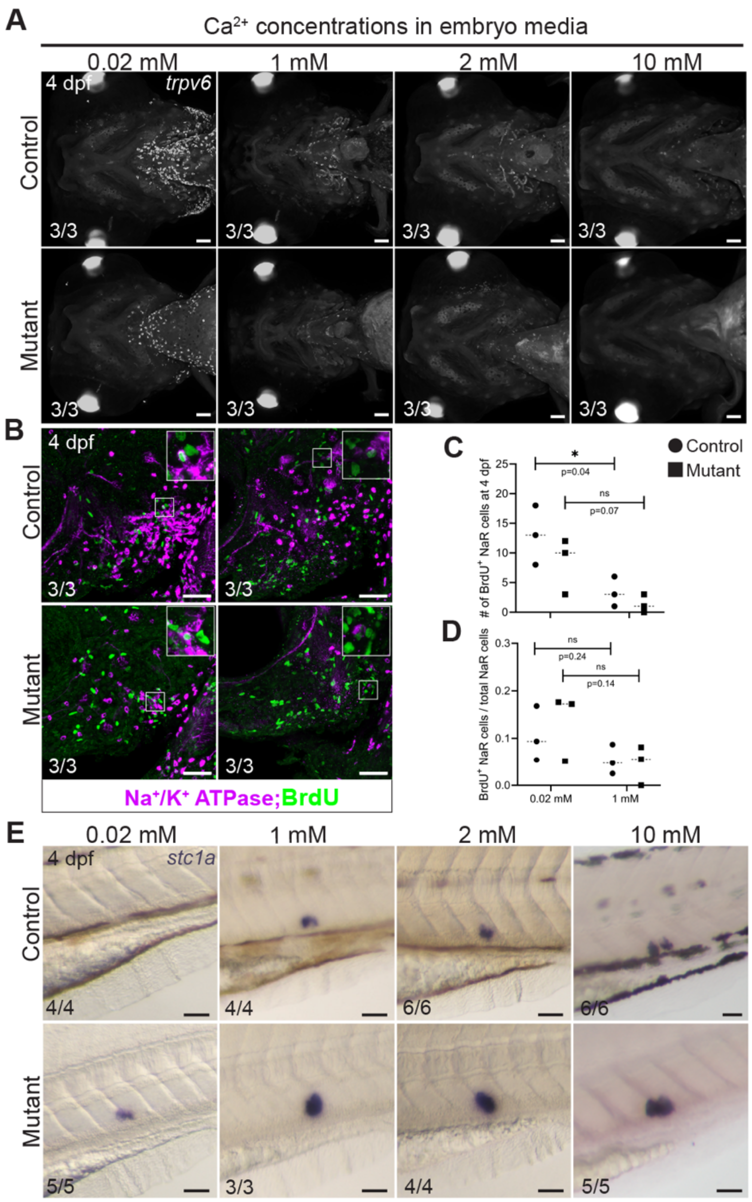

Fig. S5. *sox10* mutants still sense and respond to ambient calcium levels. (A) Fluorescent in situ hybridization confirms clear increases and decreases in the number of *trpv6*+ cells in both control and mutant larvae raised to 4 dpf in high (2 or 10 mM) or low (0.02 mM) environmental calcium, respectively, compared to standard embryo media (1 mM). **(B)** BrdU stain demonstrates increased NaR cells proliferation in both controls and mutants when raised in low Ca^{2+} medium. **(C-D)** Quantification of $BrdU^{+}$ NaR cells (C) and ratios of the $BrdU^{+}$ NaR cells to the total NaR cells (D) in C. **(E)** *stc1a* mRNA levels are reduced in both control and mutant larvae raised to 4 dpf in low environmental Ca^{2+} compared to those kept in standard medium, though transcription is not shut off completely in mutants. Levels appear marginally increased in controls raised in high Ca^{2+} but are not overtly changed in mutants. Scale bars: 50 µm.

Development • Supplementary information

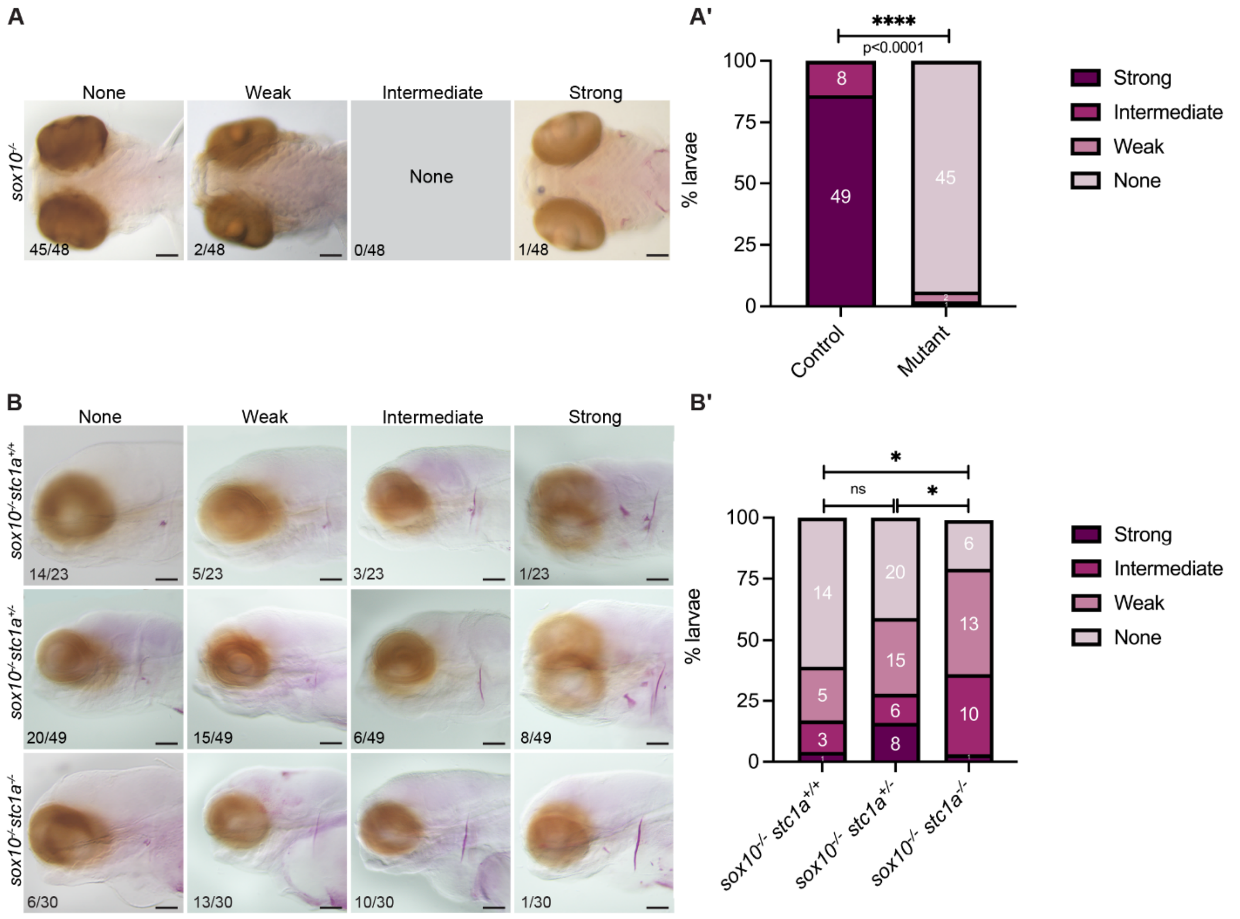

Fig. S6. Spectrum of mineralization intensities in *sox10* mutants at 4 dpf. (A-A') By Alizarin red staining, 92% (45 of 48) of *sox10* mutants imaged at 4 dpf showed a complete lack of mineralization. The remaining 8% had either faint (classified as 'weak'; 2 of 40) or strong (1 of 40) Alizarin staining. All controls were classified as intermediate or strong. There is significantly less mineralization in the mutants compared to controls (Chi-square; $p<0.0001$, df=3). **(B-B')** Partial genetic reduction of *stc1a* improved mineralization in *sox10* mutants, but a more significant improvement (Chi-square; $p=0.0206$, df=3) was observed upon complete loss of *stc1a*, as 83% of the double mutant larvae (24 out of 30) had some mineralization. The incidence of faint Alizarin staining in *sox10*$^{-/-}$; *stc1a*$^{+/+}$ larvae produced by incrossing double heterozygotes was higher than in the single *sox10* mutants quantified in A, possibly due to genetic background effects. Classes: 'strong': well-stained opercle, cleithrum and teeth; 'intermediate': well-stained cleithrum, no opercle or teeth; 'weak': faint cleithrum, no opercle or teeth; 'none': no Alizarin red staining. Scale bars: 100 μm.

Table S1. DESeq2 output of *sox10* control versus mutant RNAseq analysis

Available for download at
https://journals.biologists.com/dev/article-lookup/doi/10.1242/dev.204357#supplementary-data

Development • Supplementary information

Table S2. Select DESeq2 results for osteoblast and mineralization markers and endocrine components involved in calcium regulation.

	Symbol	Gene name	p-adjusted	log_2 FoldChange
Endocrine components	*stc1l*	stanniocalcin 1, like	0.0076	2.1824
	nkx2.4a	NK2 homeobox 4a	0.1681	0.6429
	fgf23	fibroblast growth factor 23	0.2397	1.9880
	thraa	thyroid hormone receptor alpha a	0.6794	0.2953
	duox2	dual oxidase 2	0.6840	0.8560
	calca	calcitonin-related polypeptide, alpha	0.7374	0.3259
	tg	thyroglobulin	0.8482	0.8245
	thrb	thyroid hormone receptor beta	0.8570	0.4827
	pth2ra	parathyroid hormone 2 receptor a	0.9111	0.3337
	oxt	oxytocin	0.9245	0.3541
	oxtra	oxytocin receptor a	0.9307	0.9113
	tshr	thyroid stimulating hormone receptor	0.9484	0.5776
	pth1a	parathyroid hormone 1a	0.9745	1.0061
	dio2	iodothyronine deiodinase 2	0.9888	0.3971
	dio1	iodothyronine deiodinase 1	0.9942	-0.4540
	pth1b	parathyroid hormone 1b	0.9970	-0.9576
	oxtrb	oxytocin receptor b	0.9987	-0.4907
	pth4	parathyroid hormone 4	0.9987	-0.3412
	slc5a5	solute carrier family 5 member 5	0.9987	-0.2700
	calcrlb	calcitonin receptor-like b	0.9987	-0.0422
	calcrla	calcitonin receptor-like a	0.9987	0.0401
	calcr	calcitonin receptor	0.9987	0.1055
	calcrl2	calcitonin receptor-like 2	0.9987	0.1180
	pth3r	parathyroid hormone 3 receptor	0.9987	0.1869
	pth2	parathyroid hormone 2	0.9987	0.2558
	pth2rb	parathyroid hormone 2 receptor b	0.9987	0.2574
	duox	dual oxidase	0.9987	0.2611
	thrab	thyroid hormone receptor alpha b	0.9987	0.3488
	casr	calcium-sensing receptor	NA	0.3260
Osteoblast markers	*sparc*	secreted protein, acidic, cysteine-rich (osteonectin)	0.0006	-0.7883
	col1a1a	collagen, type I, alpha 1a	0.0010	-0.8014
	col1a1b	collagen, type I, alpha 1b	0.0202	-0.8215
	col1a2	collagen, type I, alpha 2	0.0049	-0.7532
	col10a1a	collagen, type X, alpha 1a	0.3905	0.6739
	sp7	Sp7 transcription factor	0.1121	0.8305
	entpd5a	ectonucleoside triphosphate diphosphohydrolase 5a	0.9439	0.4841
	enpp1	ectonucleotide pyrophosphatase/phosphodiesterase 1	0.9568	-0.2502
	runx2b	RUNX family transcription factor 2b	0.9987	-0.3106
	phex	phosphate regulating endopeptidase homolog, X-linked	0.9987	-0.1811
	alpl	alkaline phosphatase, biomineralization associated	0.9987	-0.1600
	phospho1	phosphoethanolamine/phosphocholine phosphatase 1	0.9987	0.1417
	bglap	bone gamma-carboxyglutamate (gla) protein	0.9987	0.2994
	spp1	secreted phosphoprotein 1	0.9987	0.3694
	runx2a	RUNX family transcription factor 2a	NA	-0.1062

Development • Supplementary information

Table S3. In situ probe primers

Gene	Primers (5'→ 3')	Linearization Enzyme	RNA polymerase
trpv6	GGCAACGACCACACCATAAA<br>TGGGCCATTATGTCATTGCG	EcoRV	Sp6
col10a1a	ACCAGCCTTACTCCGTGAAA<br>GGCTCACCTTTCTGACCAGT	EcoRV	Sp6
stc1a	ATGCTCCTGAAAAGCGGATTTCTT<br>AGGACTTCCCACGATGGAGCGTTT	EcoRV	Sp6
igfbp5a	AGTTTGTATGCTCTGGTGGC<br>CCATTAAAGTCAGTGCCCGG	EcoRV	Sp6
phospho1	CCGCTTCCTGATGTTCTTCG<br>GTCCTCACCACTCTTCCAGG	EcoRV	Sp6
sparc	CTTCTTCCTGTTCTGCCTCG<br>ATAAGAGGAGCACGCAGAGG	EcoRV	Sp6
spp1	CCACGCCAACAGAATCGAAT<br>TCTCCTGGCTTTCTGTGCTT	EcoRV	Sp6
runx2a	CGCGTGTGTTTGTTTGTTCC<br>CATGGTGGTCTGGCGTAAAC	EcoRV	Sp6
runx2b	CGGTGAAGATGAACGACGTG<br>GCCAGGGAAAGGACTCAAGT	XbaI	T7

Table S4. rt-PCR primers

Gene	Primers (5' → 3')	Product size (bp)	Conditions*
stc1a	CACGGTTCTCATCCAACACC<br>GCGCTTAATGGTCTGGAACA	245	15s/30s/30s 56°C<br>30x
spp1	CAGCAAGCAGTTCAGAGAGC<br>CTGCCTCCTCAGTGTCATCT	162	15s/30s/30s 56°C<br>30x
sparc	TCCAACTGAAGAGGAGCCAG<br>ATAAGAGGAGCACGCAGAGG	164	15s/30s/30s 56°C<br>30x
enpp1	GTGCTTCTTCTTGGCTTGCT<br>CAGGTGCCTTCATTTACGCA	167	15s/30s/30s 56°C<br>28x
entpd5	CGCTACCGTGCAATCAATCA<br>TGGAAGCTCAACTGGGTCTT	195	15s/30s/30s 56°C<br>28x
phex	CTCTGTCTAGGCCCACACTG<br>TATCTCCACTTCTCACGGGC	226	15s/30s/30s 56°C<br>28x
phospho1	TTGCCCGTCTCTTACACTGT<br>GTCCTCACCACTCTTCCAGG	142	15s/30s/30s 56°C<br>30x
fgf23	ACATCCACCTTCAGACCACT<br>TCCTCCTGTATCCATGCACA	185	15s/30s/30s 56°C<br>30x
alpl	AGTTTCCAGAGCAAGAGAAGC<br>TCTGTCCACTCAACTGACCC	189	15s/30s/30s 56°C<br>30x
trpv6	CAGGCATGTACTTCCGCAAA<br>ATCCTGAGCCAGCAAGAAGT	227	15s/30s/30s 58°C<br>35x
eef1g	TCGTCTGAAGATTGCGAGTG<br>ACCCTGGTAAGCTGGAACCT	109	15s/30s/30s 56°C<br>25x

*Format: 15s denaturation, 30s annealing, 30s elongation, annealing temperature, # cycles

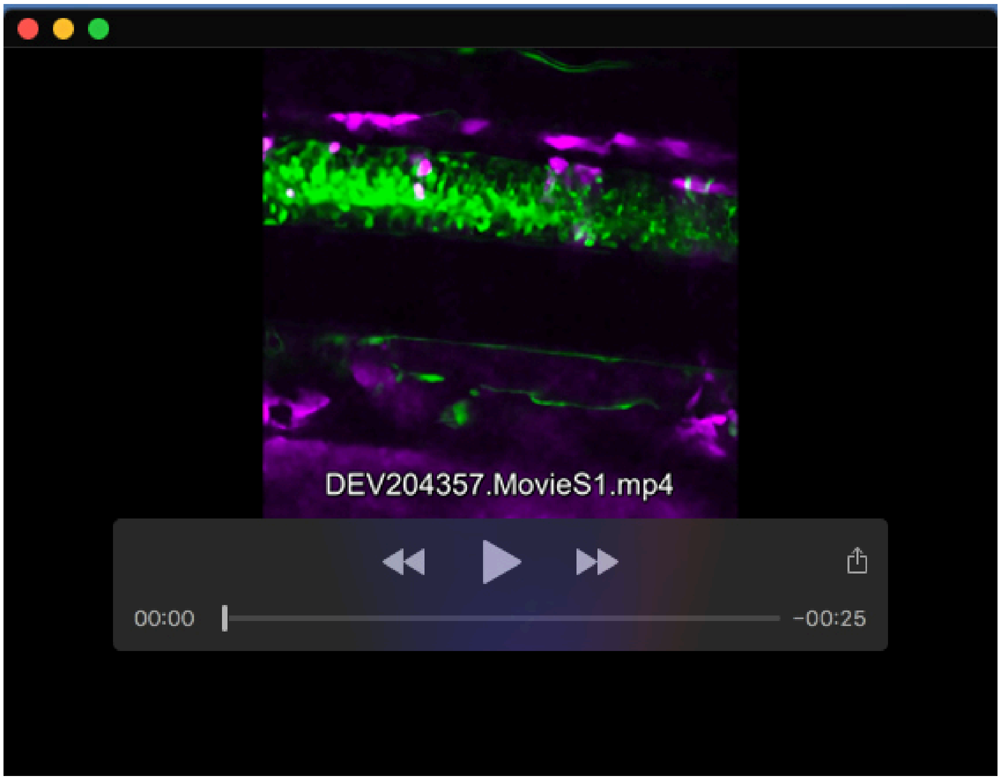

Movie 1. Dynamic and transient interaction between *sox10*[+] neural crest lineage cells and the Corpuscles of Stannius. A maximum projection of a control zebrafish time-lapse between 48 and 96 hpf demonstrating an interaction between the neural crest lineage cell, labeled by *SOX10:Cre; actb2*:BFP>DsRed in magenta and the Corpuscles of Stannius, labeled by *Tp1:VenusPEST* in green.
