# Supplementary material for: Sox10 is required for systemic initiation of bone mineralization
Source: Development. 2025 Jan 20;152(2):dev204357. doi: 10.1242/dev.204357 (PMC11833171; doi:10.1242/dev.204357)
Supplement: Supplementary file 1 [file develop-152-204357_review_history.pdf]

Development

Sox10 is required for systemic initiation of bone mineralization

Stefani Gjorcheska, Sandhya Paudel, Sarah McLeod, David Paulding, Louisa Snape, Karen Camargo Sosa, Cunming Duan, Robert Kelsh and Lindsey Barske
DOI: 10.1242/dev.204357

Editor: James Briscoe

Review timeline
Original submission: 28 August 2024
Editorial decision: 8 October 2024
First revision received: 9 December 2024
Accepted: 19 December 2024

Original submission

First decision letter

MS ID#: dev.204357

MS TITLE: Sox10 is required for systemic initiation of bone mineralization

AUTHORS: Stefani Gjorcheska, Sandhya Paudel, Sarah McLeod, David Paulding, Louisa Snape, Karen Camargo Sosa, Cunming Duan, Robert Kelsh and Lindsey Barske

I have now received all the referees' reports on the above manuscript, and have reached a decision. The referees' comments are appended below, or you can access them online: please go to:

As you will see, the referees express considerable interest in your work, but have some criticisms and recommend a revision of your manuscript before we can consider publication. The revisions and clarifications the referees request appear to be straight forward to address and should strengthen your study. If you are able to revise the manuscript along the lines suggested, I will be happy to receive a revised version of the manuscript. Your revised paper will be re-reviewed by one or more of the original referees, and acceptance of your manuscript will depend on your addressing satisfactorily the reviewers' major concerns. Please also note that Development will normally permit only one round of major revision. If it would be helpful, you are welcome to contact us to discuss your revision in greater detail. Please send us a point-by-point response indicating your plans for addressing the referees' comments, and we will look over this and provide further guidance.

Please attend to all of the reviewers' comments and ensure that you clearly highlight all changes made in the revised manuscript. Please avoid using 'Tracked changes' in Word files as these are lost in PDF conversion. I should be grateful if you would also provide a point-by-point response detailing how you have dealt with the points raised by the reviewers in the 'Response to Reviewers' box. If you do not agree with any of their criticisms or suggestions please explain clearly why this is so.

Reviewer 1

Advance summary and potential significance to field

© 2024. Published by The Company of Biologists under the terms of the Creative Commons Attribution License (https://creativecommons.org/licenses/by/4.0/).

This elegant study by Gjorcheska et al reveals an unexpected role of trunk neural crest cells in limiting the embryonic growth of the Corpuscle of Stannius in zebrafish, whose normal function is to limit calcium absorption systemically. This is turn helps to elevate absorption of calcium from the water through ionocytes to stimulate the initiation of bone formation in the embryo. I found this study highly novel as it shows a non-cell-autonomous role of neural crest cells in restricting growth of an endocrine organ during embryonic stages, and how this is important to coordinate the first wave of mineralization in vertebrates. This is a nearly flawless study with well-presented and conceived experiments, including rigorous use of quantification and statistics. The findings are sound and the model is clearly supported. It was also a well written paper with caveats and implications carefully considered throughout. I have only a few minor suggestions to address.

1. Line 245: It is stated that the differences between Ca++ rescue of trpv6 versus sox10 mutants reflects a "more complex etiology". Could this be because ionocytes in the absence of trpv6 can still take up some calcium, while in sox10 mutants the ionocytes themselves are depleted? This would be a discussion point rather than belonging in the results section.

2. Lines 341-342. The first sentence of the Discussion, "This study challenges the decades-old paradigm that Sox10 is not required for skeletal development", seems over-exaggerated and wholly unnecessary. Its unclear what decades-old paradigm is being referred to. The novelty of the current study, in particular the non-cell-autonomous role of embryonic neural crest in restricting growth of an endocrine organ for a developmental transition, is interesting in and of itself and should be the main focus and take-home point of the paper, not taking down a straw-man paradigm of how Sox10 may function.

3. It would be interesting to know the identity of the trunk neural crest cells that surround the Corpuscles of Stannius. In situs for neural progenitors (neurog1), DRGs (elavl4, isl2b), and glia (sox10) could be done in wild types to clarify. These and likely other markers are all expressed in trunk crest derivatives by 3 days or earlier.

4. More of a discussion point, but do the authors have any clues from the literature or existing sequencing datasets of what the inhibitory signals from the trunk crest to Corpuscle of Stannius might be? No new experiments are needed.

5. Fig. 6E. At 168 hpf, the mutant Tp1+ cells appear to be much bigger than the control Tp1+ cells. Are these images at the same magnification? If so, are cells in mutants greatly enlarged rather than there being more cells? Along these lines, cell counts should be performed rather than measuring the volume of CS. Also in the legend, the phrase "Only the right CS, closest to the lens, was measured" is unclear. Does lens refer to the lens of the eye or the lens of the microscope? If lens of the eye, shouldn't the left CS be closer to the lens since anterior is to the right? Or if these are the CS on the left and right sides of the embryo, that appear superimposed in the confocal projection, this should be better clarified.

Reviewer 2

Advance summary and potential significance to field

Gjorcheska et al., report a novel phenotype for the loss of the transcription factor Sox10, which is the lack of bone mineralization in zebrafish. They make the discovery that sox10 mutants have a calcium deficiency due to lack of trpv6+ ionocytes, and further show that this is likely because of an upregulation of the anti-hypercalcemic hormone stanniocalcin. Stc1a is produced in Corpuscles of Stannius, which is abnormally large in sox10 mutants, and lack a population of sox10-derived cells that may interact with it. Overall, the manuscript contains novel data on non-autonomous roles of neural crest cells in bone mineralization and is well written. The rescue of mineralization with the double mutants is quite elegant.

Comments for the author

I have a few comments to strengthen the manuscript:

© 2024. Published by The Company of Biologists under the terms of the Creative Commons Attribution License (https://creativecommons.org/licenses/by/4.0/).

The authors show based on rt-PCR that the total trpv6 expression is not changed in sox10 mutant embryos, but rather the number of NaR cells is dramatically decreased. However, trpv6 expression is relatively specific to NaR ionocytes and expression does not seem different in the in situs in Figure 4 raising the question of why total trpv6 is normal. Ionocytes are known to regulate ion channel transcription in different physiological contexts. Do the remaining NaR cells upregulate trpv6 expression in these mutants? Are the images adjusted to the same brightness and contrast levels? A quantification of trpv6 expression in these in situs would be informative as to how these cells are responding in the mutants.

When counting the number of trpv6+ cells, are the authors distinguishing from skin and gill cells or counting only the ones in the skin? Authors should specify it.

In Figure 6E control images, the authors state that the Sox10-derived cells were harder to find over time, and therefore likely turn off the gene. However, as far as I can tell the experiment uses a ubiquitous driver for the switch line (actb2). Shouldn't fluorescence be constant in these cells instead of decreasing?

High levels of stanniocalcin block proliferation of NaR ionocytes, but NaR numbers still increase in sox10 mutants when calcium is depleted from the medium. Could this increase be due to differentiation of new NaR cells instead of proliferation of the existing ones?

Minor comments:
Results
- Line 203: differences between rt-PCR and colorimetric in situ could also be due to different sensitivity of techniques
- It is not clear how long the calcium treatment experiments were. Based on Figure 3C, I understood they started at 4hpf and maintained the same high calcium levels until 4 days post fertilization. Is this correct? It would be helpful to specify in the methods if this is the case and how often the media was changed until the embryos were fixed for further analysis.
-
Figures
- Legend for Figure 1 is missing letter (B)
- Figure 2C and C' at 7 dpf, opercle in the mutant looks smaller than control
- Figure 5 letters not bold in the legend
- Figure 5C, how the cells being counted? Is it based on DAPI and overlap with the in situ signal?
- Figure 6, dorsal aorta is not abbreviated anywhere else and abbreviation could be removed
- Scale bar values missing in Fig. 6C and 6E
- Figure S1: are all scale bars 100um?

Methods
- Reference in line 450 appears misspelled
- Imaging and image analysis methods: please provide information on the objective lenses and fluorophores used

Reviewer 3

Advance summary and potential significance to field

This manuscript describes a previously unknown function of Sox10 in bone mineralization at early stages of zebrafish development. In a straightforward approach, the authors show that cranial bone mineralization is seriously delayed in two different sox10 mutants, and that this delay is due to impaired calcium mobilization by a decreased specific ionocyte subpopulation. This decrease is ultimately caused by an increase in the expression of stanniocalcin (Stc1a) in the corpuscles of Stannius, which appear to be in close contact with Sox10-expressing cells at around 50 hpf. Mutation of the stc1a gene is able to at least partially rescue the sox10 bone phenotype in double mutants, thus supporting the conclusion.

© 2024. Published by The Company of Biologists under the terms of the Creative Commons Attribution License (https://creativecommons.org/licenses/by/4.0/).

Comments for the author

I have only some minor points to address in this manuscript:

1) Figure 2, legend: it seems like the (C) and (D-D") should actually be (D) and (C-C").
2) Figure 4, legend, line 945: "Ish for igfbp5a and trpv6 (C,D)" should probably be "for trpv6 and igfbp5a (C,D)", where it would be helpful to indicate "igfbp5a" on panel D
3) Lines 247-261: it is not clear in the text (and in Fig. 4) what the authors consider as NAR cells: are the igfbp5a-expressing cells, or later in the text are they the "subset of ionocytes" that are Na+/K+ ATPase-positive cells? So are the NAR cells in Figs 4C' and D' actually Na+/K+ ATPase positive?
4) Line 275: it may be useful to mention some of the other candidate endocrine factors that were tested and not found to be significantly affected

---

First revision

Author response to reviewers' comments

Response to Reviewers
Note: We have highlighted changes to the text made in response to reviewer comments in red font. We have also made minor textual changes (also marked in red font) to align with formatting guidelines and make the text meet the specified word count.

Reviewer #1

1.1. Line 245: It is stated that the differences between Ca^{++} rescue of trpv6 versus sox10 mutants reflects a "more complex etiology". Could this be because ionocytes in the absence of trpv6 can still take up some calcium, while in sox10 mutants the ionocytes themselves are depleted? This would be a discussion point rather than belonging in the results section.

Response: This is an intriguing idea. We have added a section to the discussion to consider this possibility (Lines 398-411):

"Rearing *sox10* mutant embryos in a high-Ca^{2+} medium did not improve their mineralization deficit, in contrast to prior results for similarly under-mineralized *trpv6* and *msp* mutants (Vanoevelen et al. 2011; Huitema et al. 2012). A possible explanation for this discrepancy lies in their associated NaR cell phenotypes*:* *trpv6* mutants have excess NaR cells (Xin et al. 2019), versus the depletion observed in *sox10* mutants (Fig. 4). Though *Trpv6* is the only route of free calcium ingress in ionocytes, NaR cells express two other transporters capable of moving calcium across membranes: the Na^+/Ca^{2+} exchanger (aka Ncx1b, encoded by slc8a1b) and the plasma membrane Ca^{2+}-ATPase (aka Pmca2, encoded by *atp2b2*) (Liao et al. 2007). The Na^+/Ca^{2+} exchanger in particular normally removes calcium from cells but can also work in the reverse direction depending on ion gradients (e.g. Yu et al. 1997). Given enough excess NaR cells and very high external Ca^{2+} (as in the *trpv6* mutants raised in high Ca^{2+}), it is conceivable that Ncx1b might import sufficient Ca^{2+} to initiate mineralization. This mechanism would not be available in sox10 mutants, which have almost no NaR cells at high Ca^{2+} (Fig. S5A)."

We also have edited the Results sentence in question (now Line 265-267) to read:

"The mineralization delay in the sox10 mutants may thus have a <u>different</u> etiology than other mutant lines with similar phenotypes."

1.2. Lines 341-342. The first sentence of the Discussion, "This study challenges the decades-old paradigm that Sox10 is not required for skeletal development", seems over-exaggerated and wholly unnecessary. It's unclear what decades-old paradigm is being referred to. The novelty of the current study, in particular the non-cell-autonomous role of embryonic neural crest in

© 2024. Published by The Company of Biologists under the terms of the Creative Commons Attribution License (https://creativecommons.org/licenses/by/4.0/).

restricting growth of an endocrine organ for a developmental transition, is interesting in and of itself and should be the main focus and take-home point of the paper, not taking down a straw-man paradigm of how Sox10 may function.

Response: We thank the reviewer for this perspective and have edited the sentence as follows (now Line 370):
"This study reveals a previously undescribed, indirect role for Sox10 in skeletal mineralization."

1.3. It would be interesting to know the identity of the trunk neural crest cells that surround the Corpuscles of Stannius. In situs for neural progenitors (neurog1), DRGs (elavl4, isl2b), and glia (sox10) could be done in wild types to clarify. These and likely other markers are all expressed in trunk crest derivatives by 3 days or earlier.

Response: We emphatically agree that identifying the *sox10*$^+$ cell type interacting with the Corpuscles is of great importance, and in fact have made it the primary goal of the next stage of this project. We have performed preliminary in situs and immunostaining for some of the cell types the reviewer mentioned, but without persuasive results thus far, possibly due to the small number of cells involved and the potentially dynamic nature of their interactions (see response 2.3). These *sox10*$^+$ cells also cannot be readily detected in single-cell RNAseq datasets without a more specific marker. As mentioned in the discussion, we propose that the *sox10*$^+$ cells may be Schwann cell precursors (SCPs). These cells are known to give rise to sympathetic neurons, which in turn are known to innervate the CS in adults (Wendelaar Bonga et al., 1977, Krishnamurthy and Bern, 1971) and are largely absent in sox10 mutant fish and mice (Kelsh and Eisen, 2000, Britsch et al., 2001). We are testing specific methods for depleting SCPs as an indirect strategy for identifying the CS- interacting cells, but these experiments are beyond the scope of this first report.

1.4. More of a discussion point, but do the authors have any clues from the literature or existing sequencing datasets of what the inhibitory signals from the trunk crest to Corpuscle of Stannius might be? No new experiments are needed.

Response: This is another major goal of our ongoing work. Published studies have implicated numerous intercellular signaling pathways – including FGF, Wnt, and Notch – in the initial budding of the CS from the pronephros. However, this process appears to occur normally in *sox10* mutants, with the glands only increasing in size at later stages. Which pathways govern their growth post- budding is not known. However, the strong activity of the Notch *Tp1:VenusPEST* and *her6:mCherry* reporters at these later stages indicates that this pathway may be involved.

We have added the following to the discussion (Lines 459-468):

"Formation of the Corpuscles of Stannius from the pronephros depends on key signaling pathways such as FGF, Wnt, and Notch. However, the regulatory mechanisms that govern gland size after their initial budding remain unclear. scRNAseq data (Sur et al., 2023) and supporting studies (Drummond et al., 2017, Naylor et al., 2018, Klingbeil et al., 2022) indicate that the CS continue to express components of these signaling pathways at 55-120 hpf, after budding is completed. Moreover, live-imaging experiments (Fig. 6) confirm active Notch signaling in fully formed Corpuscles, as evidenced by the reporters *Tp1:VenusPEST* and *her6:mCherry*. It is plausible that sox10+ cells communicate with the CS through one or more of these pathways, with disrupted signaling affecting CS cell proliferation and stanniocalcin production in mutants."

1.5. Fig. 6E. At 168 hpf, the mutant Tp1+ cells appear to be much bigger than the control Tp1+ cells. Are these images at the same magnification? If so, are cells in mutants greatly enlarged rather than there being more cells? Along these lines, cell counts should be performed rather than measuring the volume of CS.

Response: We noticed this as well, though the magnification is the same in controls and mutants. [n=5]. We attempted to count Venus$^+$ cells by in z-stacks collected from live-imaged embryos, but the cytoplasmic reporter made it difficult be confident about cell boundaries. We

© 2024. Published by The Company of Biologists under the terms of the Creative Commons Attribution License (https://creativecommons.org/licenses/by/4.0/).

then tried fixing the samples and performing a nuclear stain before imaging/counting; however, the Venus protein poorly survived the fixation. Our counts of *stc1a*$^{+}$ cells (Fig. 5C) do confirm that the mutant CS contain more cells, but it is possible that the cells themselves are also bigger.

Lines 441-442: "The cells themselves may also be larger (compare CS cell size at 168 hpf in Fig. 6E)."

1.6. Also in the legend, the phrase "Only the right CS, closest to the lens, was measured" is unclear. Does lens refer to the lens of the eye or the lens of the microscope? If lens of the eye, shouldn't the left CS be closer to the lens since anterior is to the right? Or if these are the CS on the left and right sides of the embryo, that appear superimposed in the confocal projection, this should be better clarified.

Response: We regret this lack of clarity. We were referring to the lens of the microscope. The gland that was measured was the one on the right side of the embryo, as the larvae were positioned on their right side. The text has been updated to reflect this (Lines 1104-1105).

Reviewer #2

2.1 The authors show based on rt-PCR that the total trpv6 expression is not changed in sox10 mutant embryos, but rather the number of NaR cells is dramatically decreased. However, trpv6 expression is relatively specific to NaR ionocytes and expression does not seem different in the in situs in Figure 4 raising the question of why total trpv6 is normal. Ionocytes are known to regulate ion channel transcription in different physiological contexts. Do the remaining NaR cells upregulate trpv6 expression in these mutants? Are the images adjusted to the same brightness and contrast levels? A quantification of trpv6 expression in these in situs would be informative as to how these cells are responding in the mutants.

Response: We agree that this discrepancy between in situ and rt-PCR results is difficult to explain, but we did not wish to exclude the latter, given that it held up in 4 independent runs and in the new RNAseq data. We confirmed that our rt-PCR primers capture all three isoforms, and that the probe is complementary to a shared part of the gene. The controls and mutants presented in Figs. 4 and 7 are adjusted to the same brightness and contrast levels. We collected additional high-magnification images but observed similar variable staining intensity across controls and mutants. We note, though, that the in situ method we use is not designed to quantitatively detect per-cell changes in gene transcription. Another possibility is that there may be broad, low-level trpv6 expression in the brain (Lin et al., 2011), which would be detected by rt-PCR but not included in our counts. Though we are not equipped to perform a quantitative in situ at present, we have added text to acknowledge the possibility that the few remaining NaR cells in mutants might be upregulating trpv6 on a per-cell level.

Lines 278-283: "Though rt-PCR revealed no overt change in whole-body *trpv6* levels (Fig. 4B), possibly due to low-level expression elsewhere in the body (Lin et al., 2011), we did detect significant
…. Whether per-cell cell *trpv6* transcription is also affected remains to be determined."

2.2. When counting the number of trpv6+ cells, are the authors distinguishing from skin and gill cells or counting only the ones in the skin? Authors should specify it.

Response: We now clarify in the Methods which counts include predominantly gill or skin ionocytes, or both (Lines 629-635). Most of the ionocytes in the ventral images (Fig. 4) were in the gills, whereas the lateral images (Fig. 7) also included skin ionocytes. We had to take lateral images for the epistasis analysis because the cardiac edema of the double mutants and stc1a single mutants prevented capturing quality ventral images.

2.3. In Figure 6E control images, the authors state that the Sox10-derived cells were harder to find over time, and therefore likely turn off the gene. However, as far as I can tell the experiment uses a ubiquitous driver for the switch line (actb2). Shouldn't fluorescence be constant in these cells instead of decreasing?

© 2024. Published by The Company of Biologists under the terms of the Creative Commons Attribution License (https://creativecommons.org/licenses/by/4.0/).

Response: This has also been of concern to us, because we agree with Reviewer 2 that Cre-mediated recombination should result in permanent DsRed expression. We consider two potential contributors:

First, we and others have anecdotally observed that this *sox10*:Cre line is mosaic and does not convert all neural crest cells in an individual fish. Though this does not explain the decreasing frequency of interaction over time, it does help to explain the inconsistency.

Second, the interaction may be more dynamic and transient than originally appreciated. With the Cre line, we see a close interaction (apparent contact) between *sox10*$^{+}$ lineage cells and the CS in 47% (8 out of 17) of larvae at 50 hpf, 25% (5 out of 19) at 3 dpf, and hardly any at 4 and 7 dpf (1 out of 14 and 1 out of 8, respectively). With the *sox10*:DsRed line, we see interactions in 65% of larvae at 50 hpf, possibly due to more uniform expression or stronger signal detection from this transgene. This may indicate a transient or dynamic relationship between the *sox10*$^{+}$ lineage cells and the glands, and that the pivotal signals may be exchanged at 2-3 dpf. This interpretation is supported by additional live imaging experiments that have captured transient and dynamic interactions.
We include an additional supplemental movie (S1) to illustrate this in the revision, and have modified the Results to include the following (Lines 333-339):
"We detected close physical interactions between the CS and *sox10*:DsRed+ or *SOX10*:Cre-traced cells at 50 and 72 hpf (Fig. 6C), after the glands had fully formed. Direct contact was observed in 47% (8 out of 17) and 65% (9 out of 14) of DsRed^{+} and Cre^{+} embryos, respectively, at 50 hpf (see Movie S1), but only in ~25% (5 out of 19) at 72 hpf. Lineage-traced crest remained in the general vicinity of the CS up to 7 dpf but were much less likely to be captured in close contact (Fig. 6D-E), suggesting the interactions may be transient and/or dynamic."

2.4. High levels of stanniocalcin block proliferation of NaR ionocytes, but NaR numbers still increase in *sox10* mutants when calcium is depleted from the medium. Could this increase be due to differentiation of new NaR cells instead of proliferation of the existing ones?

Response: This is a valid possibility. To check whether NaR cells are proliferating in *sox10* mutants raised in low calcium, we exposed them to BrdU shortly before fixing, then quantified BrdU+ NaR cells. Double-positive cells were observed in both mutants and controls, more frequently in larvae raised in low vs. normal calcium (new panels B-D added to Fig. S5; new Method, Lines 582-593). This supports that the NaR cell increase seen in the mutants under low calcium occurs at least in part through proliferation, as in controls.

2.5. Line 203: differences between rt-PCR and colorimetric in situ could also be due to different sensitivity of techniques

Response: The reviewer makes a very good point. We now note this possibility at Lines 205-206.

2.6. It is not clear how long the calcium treatment experiments were. Based on Figure 3C, I understood they started at 4hpf and maintained the same high calcium levels until 4 days post fertilization. Is this correct? It would be helpful to specify in the methods if this is the case and how often the media was changed until the embryos were fixed for further analysis.

Response: We have edited the method section to include greater detail on the calcium treatment (Lines 549-551):

"Embryos were placed into the different treatment media at 4 hpf and kept there until 4 dpf. A stock of each treatment media was prepared on the first day, and the medium in each dish was replaced daily."

2.7. Legend for Figure 1 is missing letter (B)

Response: This has been fixed.

2.8. Figure 2C and C' at 7 dpf, opercle in the mutant looks smaller than control.

© 2024. Published by The Company of Biologists under the terms of the Creative Commons Attribution License (https://creativecommons.org/licenses/by/4.0/).

Response: We see this level of variability in both control and mutant fish. It may be in part due to the angle and the size of the fish. We have replaced them with better matched pictures.

2.9. Figure 5 letters not bold in the legend.

Response: This has been fixed.

2.10. Figure 5C, how the cells being counted? Is it based on DAPI and overlap with the in situ signal?

Response: We counted stc1a$^+$ cells in Imaris by adding Spots into each cell-like sphere within the gland. We did test the use of a nuclear stain, but this actually made counting more challenging due to cell overlapping.

2.11. Figure 6, dorsal aorta is not abbreviated anywhere else and abbreviation could be removed.

Response: We have removed the abbreviation and labeled the structure with its full name in Fig. 6A and C, for anatomical reference.

2.12. Scale bar values missing in Fig. 6C and 6E

Response: This has been fixed.

2.13. Figure S1: are all scale bars 100um?

Response: Yes, we have confirmed that all scale bars are the same size.

2.14. Reference in line 450 appears misspelled

Response: Thank you for catching this; we have corrected the citation error in lines 507 and 1008.

2.15. Imaging and image analysis methods: please provide information on the objective lenses and fluorophores used.

Response: We have edited this method section as follows (Lines 619-626):
"Skeletal stains, brightfield images and colorimetric in situs were imaged on a Zeiss SteREO Discovery.V8 (1.5x objective and 8x magnification) or Zeiss Axioimager.Z1 (10x objective) microscopes, whereas fluorescent in situs, fluorescent bone stains, live transgenic fish, and immunostained specimens were imaged on a Nikon C2 confocal. The high magnification confocal images were captured with a 40x objective and 2.5x scanning zoom, while the lower magnification whole-body and ventral head pictures were taken with a 10x or 20x objective. Images of red fluorophores and fluorescent proteins were pseudocolored magenta for easier interpretation by the color-blind."

Reviewer #3

3.1. Figure 2, legend: it seems like the (C) and (D-D") should actually be (D) and (C-C").

Response: Thank you for catching this error; we have switched their order in the legend and corrected the panel letters.

3.2. Figure 4, legend, line 945: "Ish for igfbp5a and trpv6 (C,D)" should probably be "for trpv6 and igfbp5a (C,D)", where it would be helpful to indicate "igfbp5a" on panel D

Response: We have amended this legend and figure as suggested.

3.3. Lines 247-261: it is not clear in the text (and in Fig. 4) what the authors consider as NAR cells: are the igfbp5a-expressing cells, or later in the text are they the "subset of ionocytes" that are Na+/K+ ATPase-positive cells? So are the NAR cells in Figs 4C' and D' actually Na+/K+ ATPase

© 2024. Published by The Company of Biologists under the terms of the Creative Commons Attribution License (https://creativecommons.org/licenses/by/4.0/).

positive?

Response: We regret this lack of clarity. *trpv6* and *igfbp5a* are both specific markers of NaR cells. Several ionocyte classes (Na^+/K^+-ATPase Rich (NaRs), H^+-ATPase-rich (HR) and Na^+- Cl^- cotransporter-expressing (NCC)) express Na^+/K^+ ATPase, but only NaRs also co-express trpv6 and *igfbp5a*. We have edited the text to clarify that the NaR cells are a subset of the cells marked by the ATPase antibody.

Lines 273-27276: "Immunostaining for Na^+/K^+ ATPase combined with the *SOX10*:Cre lineage label (driven by a human neural crest-specific enhancer) in otherwise wild-type fish confirmed that ionocytes (including the NaR subset) do not derive from neural crest (Fig. 4A), but rather are ectodermal (Janicke et al., 2007)."

Fig. 4A legend: "Immunostaining of a 3 dpf *SOX10*:Cre; ef1a: *DsRed>GFP* larva with an antibody against the Na^+/K^+ ATPase pump confirms that ionocytes do not derive from neural crest."

3.4. Line 275: it may be useful to mention some of the other candidate endocrine factors that were tested and not found to be significantly affected

Response: The new bulk RNAseq data included in this revision shows non-significant differences in many other calcium-regulating hormones, including *fgf23*, *calca*, and *pth1a* (Table S2). We also include a new set of experiments describing our early foray into thyroid gland development and function, which was based on the presence of neural crest cells in this organ in mammals. We have added new paragraphs in the results and methods as well as a new supplemental figure (new Fig. S3) describing this work.
Lines 211-228 (Results):
"Mineralization deficit not associated with thyroid malfunction
Several patients with *SOX10*-associated Kallmann syndrome have been reported to exhibit altered thyroid function and reduced bone mineral density (Wang et al., 2018, Chen et al., 2020, Wakabayashi et al., 2021). While the mammalian thyroid gland primarily originates from pharyngeal endoderm (De Felice and Di Lauro, 2004), some of its supporting cells derive from neural crest (Le Douarin and Le Lievre, 1970). The endoderm-derived thyroid follicles secrete thyroid hormones (T4 and T3), which bind to receptors in osteoblasts and activate signaling pathways essential for bone mineralization and growth (Bassett and Williams, 2016). Based on this connection, we initially hypothesized that loss of *sox10* may deplete a neural crest population important for the development of the thyroid gland, thereby reducing thyroid hormone levels and bone mineralization. To test this, we incubated *sox10* mutants and sibling controls in media containing T3 doses ranging from 50 to 600 $\mu g\ L^{-1}$ (Fig. S3). Treated controls showed a dose-dependent reduction in melanocyte coverage (Fig. S3B), indicative of hyperthyroidism (McMenamin et al., 2014). However, no rescue of mineralization was observed in 4 dpf mutants at any T3 dose (Fig. S3C). Fluorescent in situ hybridization showed no structural abnormalities in mutant thyroid follicles (Fig. S3A,D), located along the ventral aorta (Porazzi et al., 2009). Further, no significant differences in genes associated with thyroid hormone synthesis or signaling were uncovered in our RNAseq dataset (Table S2). These findings suggest that the mineralization deficit in zebrafish *sox10* mutants is likely not due to thyroid dysfunction."

Lines 554-562 (Methods):
"Thyroid hormone treatment
Triiodothyronine (T3) (T2877, Sigma Aldrich) was added to embryo media at approximately 32 hours post-fertilization (hpf), coinciding with the onset of thyroid gland activity. T3 was administered at concentrations of 50, 100, 300, and 600 μgl^{-1} in 30 ml of embryo media, following established protocols (Zada et al., 2016, Walter et al., 2019). The media was refreshed daily until 4 dpf, after which skeletal staining was performed to assess mineralization. Each treatment was conducted in four independent experiments, with at least six *sox10* mutants and six wild-type sibling controls included per group. This ensured sufficient replicates to evaluate the effects of T3 on skeletal development."

© 2024. Published by The Company of Biologists under the terms of the Creative Commons Attribution License (https://creativecommons.org/licenses/by/4.0/).

Second decision letter

MS ID#: dev.204357R1

MS TITLE: Sox10 is required for systemic initiation of bone mineralization

AUTHORS: Stefani Gjorcheska, Sandhya Paudel, Sarah McLeod, David Paulding, Louisa Snape, Karen Camargo Sosa, Cunming Duan, Robert Kelsh and Lindsey Barske

I am happy to tell you that your manuscript has been accepted for publication in Development, pending our standard publication integrity checks.

Comments from the Reviewers:

Reviewer 1: The authors have satisfactorily addressed all my concerns. This is an excellent study that should be of wide interest to the bone mineralization field, and shows more generally a surprising new role of neural crest cells in regulating development of the skeleton.

Reviewer 2: SUMMARY OF THE ADVANCE MADE IN THIS PAPER AND ITS POTENTIAL SIGNIFICANCE TO THE FIELD

SUGGESTIONS TO AUTHORS
The authors have performed additional experiments and addressed all of my comments.

Reviewer 3: SUMMARY OF THE ADVANCE MADE IN THIS PAPER AND ITS POTENTIAL SIGNIFICANCE TO THE FIELD

SUGGESTIONS TO AUTHORS
All my concerns have been dealt with.

© 2024. Published by The Company of Biologists under the terms of the Creative Commons Attribution License (https://creativecommons.org/licenses/by/4.0/).
